# Nucleotide sequence variants, gene expression and serum profile of immune and antioxidant markers associated with bacterial diarrhea susceptibility in Barki lambs

**DOI:** 10.1186/s12917-024-04288-1

**Published:** 2024-10-11

**Authors:** Asmaa Darwish, Eman Ebissy, Amani Hafez, Ahmed Ateya, Ahmed El-Sayed

**Affiliations:** 1 Department of Animal Health and Poultry, Animal and Poultry Production Division, Desert Research Center (DRC), Cairo, Egypt; 2 Department of Development of Animal , of Veterinary Medicine, Mansoura University, Mansoura, Egypt

**Keywords:** Barki lambs, Diarrhea, Gene expression, Immunity, Antioxidant, Nucleotide sequence variants

## Abstract

**Background:**

Despite the fact that diarrhea is more accurately described as a clinical symptom than a disease. Diarrhea is one of the most important issues in ovine medicine, particularly in lambs, and because of high morbidity and mortality rate, sluggish growth performance, and veterinary costs, it is believed to be a major source of economic loss. *Salmonella* and enterotoxigenic *Escherichia coli* are the most common and commercially significant agents responsible for diarrhea.

**Objective:**

The objective of this study was to monitor the nucleotide sequence variations, gene expression, serum inflammatory and oxidative stress biomarkers in diarrheic lambs. Another aim was to identify different pathotypes and virulence genes of *Salmonella* and *E. coli* causing diarrhea.

**Methodology:**

Blood samples were taken from 50 Barki who were diarrheal and 50 who appeared to be healthy, and then divided in 3 portions, with EDTA added to the first part for CBC, DNA and RNA extraction. The second sample received 5000 I.U. of heparin calcium, and a clean plain tube was used for the third component. The second and third sections were centrifuged to extract serum and plasma until the biochemical and immunological analysis was completed. Fecal samples were collected for bacteriological examination, and the bacteria were identified by PCR analysis. PCR-DNA sequencing was conducted for immune (*SELL, JAK2, SLC11A1, IL10, FEZF1, NCF4, LITAF, SBD2, NFKB, TNF-α, IL1B, IL6, LGALS,* and *CATH1*), antioxidant (*SOD1, CAT, GPX1, GST, Nrf2, Keap1, HMOX1*, and *NQO1*), and GIT health (*CALB1, GT*, and *MUC2*) genes in healthy and diarrheic lambs.

**Results:**

Virulent genetic markers of pathogenic characteristics of *E. coli* (astA, Vt2e (Stx2e), CFA/I, *groES* and *luxS*) and *Salmonella* (*invA*, *SopB*, *bcfC* and *avrA*) were detected in all diarrheic lambs. PCR-DNA sequencing of immune, antioxidant and intestinal health genes found eleven single nucleotide polymorphisms (SNPs) linked to either diarrhea resistance or susceptibility in Barki lambs. Transcript levels of immune, antioxidant, and GIT health (*CALB1, GT*, and *MUC2*) genes varied between healthy and diarrheic lambs. Nucleotide sequence variation of the genes under inquiry between reference sequences in GenBank and those of the animals under investigation verified all identified SNPs. Significant (*P* = 0.001) erythrocytosis, neutrophilic leukocytosis, with lymphocytopenia were observed in diarrheic lambs. Significant (*P* = 0.001) increases in serum IL-1α, IL-1β, IL-6, TNF-α (90.5 ± 1.7, 101.8 ± 1.7, 72.3 ± 6.6, 71.26 ± 4.89 Pg/ml, respectively), serum Fb, Cp, Hp, SAA (230.7 ± 12.4 mg/dl, 6.5 ± 0.07 mg/dl, 2.5 ± 0.09 g/dl, 7.4 ± 0.4 mg/L, respectively), free radicals (MDA, NO), cortisol (6.91 ± 0.18 μg/dl) and growth hormone, with significant (*P* = 0.001) decreases in serum IL-10 (81.71 ± 1.05 Pg/ml), antioxidants (CAT, GPx), insulin, triiodothyronine (T3) and thyroxine (T4) in diarrheic lambs.

**Conclusions:**

The study's findings provided credence to the theory that marker-assisted selection (MAS) could be used to predict and prevent diarrhea in Barki sheep by selecting lambs based on SNPs in genes linked to inflammation, antioxidants, and intestinal health. In order to establish an efficient management protocol and determine the most susceptible risk period for disease occurrence, gene expression profiles of the genes under investigation, pro-inflammatory cytokines and acute phase proteins may also be utilized as proxy biomarkers for lamb enteritis.

**Supplementary Information:**

The online version contains supplementary material available at 10.1186/s12917-024-04288-1.

## Background:

The Barki sheep breed, named after the Libyan province Barka, is indigenous to Egypt's northwest coastal region, where the local people depends heavily on it for their way of life [1]**.** It covers a large geographic area that stretches from Alexandria, Egypt, to the east of Libya. It rules the northwest desert of Egypt and is well renowned for having a strong capacity for adapting to the severe weather, scarcity of food, and heat stress [2]**.**

Even though diarrhea is actually a clinical symptom rather than an actual disease. In the practice of ovine medicine, diarrhea is one of the most serious problems, especially in lambs, and it is thought to be a significant source of economic loss because of mortality, slow growth rates, and veterinary expenses [3]. A variety of pathogens, including bacteria, viruses, protozoa, and intestinal parasites, are described as important agents causing diarrhea (either separately or in combination) [4]. Its complex etiology involves management, environmental, nutritional, physiological, and management-related factors. The most prevalent and economically significant agents are known to be *Salmonella* and enterotoxigenic *E. coli* (ETEC) [5, 6]. However, other bacteria, such as *Campylobacter spp.* and *Clostridium spp.*, have also been found to be the cause of diarrhea and gastrointestinal illness [7]. The diarrheic lamb is a clinical condition linked to a number of illnesses marked by diarrhea rather than a distinct medical entity. Whatever the source, abnormal fluid absorption from the intestine results in potentially fatal electrolyte imbalances [8].

*Escherichia coli (E. coli)* is a microorganism that lives in both human and animal intestines. Numerous intestinal and extra-intestinal illnesses, including meningitis in newborns, septicemia, urinary tract infections, and diarrhea, can be brought on by specific *E. coli* strains [9]. Two illnesses have been connected to two different strains of pathogenic *E. coli*. According to [10], the frequency of enteric and septicemia infections in the host affects the microorganism and physiological state of the infection. Additionally, enteric pathogenic *E. coli* isolates exhibit a variety of virulence traits that are categorized into a number of major pathotypes based on their pathogenesis [11]. Salmonellosis is typically presents as enteritis and septicemia, which can result in diarrhea and other potentially lethal consequences [12]. According to [13], *Salmonella* can infrequently cause disorders like suppurative epididymo-orchitis, arthritis, respiratory illnesses, meningitis, abortions, and stillbirths. *Salmonella* serovar, virulence, and antibiotic sensitivity are among the bacterial traits that affect how serious an infection is [14].

Observing deviations in blood parameters can aid in disease diagnosis and assess host tissue damage and infection severity, with hematobiochemical changes often linked to diarrhea [15]. According to [16], oxidative stress brought on by diarrhea is a key contributor to the tissue damage expansion. It can influence enzymatic activity, signal transcription, and gene expression, particularly that of the apoptotic gene. According to [17], Reactive oxygen species (ROS) are produced in greater amounts by Enterobacteriaceae. ROS rupture inner and outer mitochondrial membranes, release cytochrome-c, and trigger apoptosis, all of which enhance DNA fragmentation.

Acute Phase Response (APR) is a response that happens when an organism experiences acute infection, inflammation, immunological disease, trauma, or neoplasia. According to [18], this response is characterized by metabolic and systemic alterations. Determining the levels of APPs in circulation offers information about the ongoing inflammatory response because the amount of APP in plasma concentrations is related to the intensity and activity of inflammation [19]. Even at very low quantities, a class of soluble proteins known as cytokines can regulate the activity of cells and tissues. They have a brief half-life, are produced locally in response to stimuli, and can function in an endocrine, exocrine, or autocrine way [20].

Molecular genetic approaches provide promise for partially addressing the inadequacies of conventional methods used by animal breeders in their efforts to improve the health of their animals [21]. The idea of a selection criterion in genomic techniques for the development of disease resistance is modified from phenotypically expressed illness state to an allele one at the DNA level. "Marker-assisted selection" (MAS) is the name given to this selection procedure. MAS is an excellent method for selecting for genetically disease-resistant animals since it enhances selection accuracy without being impacted by environmental factors and permits selection without exposure to disease challenges [22]. One of the primary barriers to employing selection for animals resistant to disease is their exposure to pathogens. It is impractical to reject the practise since there is no guarantee that animals bred for reproduction would get humane treatment. As a result, a great deal of work has been done to find DNA markers for illness resistance, and several advantageous single nucleotide polymorphisms (SNPs) have been published [23].

To the best of our knowledge, there has not been any research on the incidence of diarrhea in Barki lambs that relies on determining the causal factor in addition to examining the relationships between pro-inflammatory cytokines, oxidative stress indicators, and genetic polymorphisms. The purpose of this study is to provide light on the many pathotypes of *Salmonella* and *E. coli* isolated from lambs that had diarrhea, as well as their virulence genes. Another goal was to evaluate the efficacy of proinflammatory cytokines, oxidative stress biomarkers, and genetic polymorphisms as potential indicators of diarrhea in Barki lambs.

## Materials and methods

### Animals and study design

A total of one hundred weaned Barki lambs of both sexes, aged three to six months, were randomly selected and weighed 18.1 ± 2.7 kg. In the research region, which included Mariut Research Station, Desert Research Center, El-Amria, Alexandria, this breed was the most prevalent. The weaned lambs were housed in semi-open shaded pens and were kept under identical conditions of housing throughout the study period. The basal diet was formulated to meet the lamb’s nutrient requirements in order to meet their energy and nutrient requirements of this age and size according to [24] recommendations. Animals fed on concentrates (14–16% protein), consist of 50% maize, 20–30% wheat bran, 10–15 soya bean meal, 10–15% cotton seed cake, 1% minerals and lime stone (900–1100 g/head/day) and hay (350–600 g/head/day). Diet was offered twice a day in the morning and evening with free access to water. Using the techniques outlined by [25]**,** a thorough clinical examination of the animals was conducted based on an assessment of the heart, lungs, rumen, and intestine as well as other vital signs. Animals were released and were divided according to health condition into control group (CG): 50 lambs that seem healthy (normal body temperatures, pulse, respiration rates, shiny eyes (no discharges), normal wet muzzle and muffle, no abnormal lung sounds on auscultation, raised head, normal posture and appetite, no diarrhea or lameness). Diarrehic group (DG): 50 lambs suffered from diarrhea, emaciation, pain, dullness, depression, hyperthermia, increased pulse and respiratory rates.

### Sampling

#### Fecal sampling

Fecal samples were collected by placing approximately 10 g of fecal material obtained during rectal retrieval into Whirl–Pak bags. Each sample was collected using an individual glove. Following the collection process, the samples were immediately sent to a central laboratory located at the Department of Microbiology, Desert Research Center. The samples were promptly transferred on the same day of collection using containers equipped with ice packs, and were immediately processed.

#### Blood sampling

Ten milliliters of blood was aspirated from the lambs of the studied groups through jugular vein puncture. Each sample was divided in 3 portions: The first portion received EDTA addition, and it was used immediately for DNA and RNA extraction as well as hematological analysis using an automated blood cell counter (Exigo eos veterinary Hematology system, Boule Medical AB, Sweden). The second sample was treated with 5000 I.U. of heparin calcium to stop the coagulation process, and the third portion was placed in a clean, plain tube (without any additives) to guarantee coagulation. The second and third sections were immediately centrifuged for 20 min at 37 ºC at 3000 r.p.m. to extract serum and plasma, respectively. Plasma and serum were kept at –80 until the immunological analysis was completed.

### Bacterial isolation

#### Sample inoculation.

Three g of each fecal sample was mixed with normal saline and centrifuged. The supernatant was discarded and the deposit was inoculated with 5 mL of buffered peptone water (Oxoid).

#### Selective enrichment and isolation of *E. coli* and *salmonellae*

The samples were inoculated into MacConkey broth for enrichment at 37°C for 24 h, the enrichments were streaked on MacConkey agar and incubated for 24 h at 37ºC. Pink coloured colonies were sub cultured on Eosin Methelene Blue (EMB) agar. Colonies producing greenish metallic sheen on EMB agar were considered as having *E. coli* [26]. In addition, various biochemical tests (oxidase negative, lactose fermentation positive, indole production positive, methyl red positive, Vogues Proskauer negative, citrate utilization negative, urease negative and catalase tests positive) were done for the confirmation of *E. coli* as proposed by [27].

To isolate *salmonellae*, 1–2 g of faeces were inoculated into 10 ml of tetrathionate broth (HiMedia) after incubated at 42°C for 48 h. A loop-full was streaked onto the surface of xylose lysine deoxycholate (XLD) (OXOID, England) and brilliant green agar (BGA) (OXOID, England) medium and incubated at 37^∘^ C for 24 to 48 h. The XLD and BGA plates were examined for the presence of *Salmonella* colonies. If growth is slight or if typical colonies of *Salmonella* were not present, the plates were re incubated for a further 18 to 24 h and reexamined for the presence of typical *Salmonella* colonies. The formation of red colonies with black centers and of pink colonies with a red zone was inspected on XLD and BGA plates, respectively [28]. Gram staining, biochemical tests and PCR amplification initiated the isolates identification.

All suspected *Salmonella* colonies were picked from the agar plates and inoculated for 24 or 48 h at 37∘ C into the following biochemical test for confirmation, triple sugar iron (TSI) test (*Salmonella* colonies produce black colonies or colonies with black centers and red medium on TSI agar) (OXOID, England), citrate test (produce blue colour), catalase positive, urease test (produce purple-red colour), lysine decarboxylase (LDC) agar (OXOID, England) test (produce purple-coloured, and indole test (produce violet-coloured colonies [29].

#### PCR-based bacterial detection

The QIAamp DNA Mini kit (Qiagen, Germany, GmbH) was used to extract DNA from fecal broth samples, with certain changes made in accordance with the manufacturer's instructions. In summary, 200 µl of the sample suspension was treated for 10 min at 56 µC with 10 µl of proteinase K and 200 µl of lysis buffer. 200 µl of 100% ethanol was added to the lysate following incubation. After that, the sample was centrifuged and cleaned in accordance with the manufacturer's instructions. The kit contained 100 µl of elution buffer, which was used to elute the nucleic acid.

The primers sequences, target genes, amplicon sizes and PCR cycling conditions, are mentioned in Table 1. *E. Coli* ATCC 25922 and *Salmonella* ATCC 14028 were the control positives, and the negative control reaction mixes contained sterile distilled water. Positive and/or negative controls for virulence genes were field samples that had previously been determined to be positive or negative by PCR for the relevant genes in the Animal Health Research Institute's Reference Laboratory for Veterinary Quality Control on Poultry Production.

**Table 1:** Primers sequences, target genes, amplicon sizes and cycling conditions

Target gene	Primers sequences	Amplified segment (bp)	Primary denaturation	Amplification (35 cycles)			Final extension	Reference
				Secondary denaturation	Annealing	Extension		
*E. coli phoA*	CGATTCTGGAAATGGCAAAAG	720 bp	94˚C 5 min	94˚C 30 s	55˚C 40 s	72˚C 45 s	72˚C 10 min	[30]
	CGTGATCAGCGGTGACTATGAC							
*chuA*	GAC GAA CCA ACG GTC AGG AT	279 bp	94˚C 5 min	94˚C 30 s	55˚C 30 s	72˚C 30 s	72˚C 7 min	[31]
	TGC CGC CAG TAC CAA AGA CA							
*yjaA*	TGA AGT GTC AGG AGA YGC TG	211 bp	94˚C 5 min	94˚C 30 s	55˚C 30 s	72˚C 30 s	72˚C 7 min	
	ATG RAG AAT GCG TTC CTC AAC							
*tspE4C2*	GAG TAA TGT CGG GGC ATT CA	152 bp	94˚C 5 min	94˚C 30 s	55˚C 30 s	72˚C 30 s	72˚C 7 min	
	CGC GYC AAC AAA GTA TTR CG							
*luxS*	ATGCCGTTGTTAGATAGCTTCA	513	94˚C 5 min	94˚C 30 s	55˚C 30 s	72˚C 30 s	72˚C 10 min	[32]
	GATGTGCAGTTCCTGCAACTTC							
*Vt2e*	CCAGAATGTCAGATAACTGGCGAC	322	94˚C 5 min	94˚C 30 s	57˚C 30 s	72˚C 30 s	72˚C 7 min	[33]
	GCTGAGCACTTTGTAACAATGGCTG							
*astA*	CCATCAACACAGTATATCCGA	110	94˚C 5 min	94˚C 30 s	55˚C 30 s	72˚C 30 s	72˚C 7 min	[34]
	GGTCGCGAGTGACGGCTTTGT							
*CFA/I*	GCTCTGACCACAATGTTGA	364	94˚C 5 min	94˚C 30 s	50˚C 40 s	72˚C 40 s	72˚C 10 min	[35]
	TTACACCGGATGCAGAATA							
*groES*	CGTGATCGTCAAGCGTAAAG	191	94˚C 5 min	94˚C 30 s	60˚C 30 s	72˚C 30 s	72˚C 7 min	[36]
	CCGTAGCCATCGTTGAAAAC							
*Salmonella invA*	GTGAAATTATCGCCACGTTCGGGCAA	284	94˚C 5 min	94˚C 30 s	55˚C 30 s	72˚C 30 s	72˚C 7 min	[37]
	TCATCGCACCGTCAAAGGAACC							
*Salmonella bcfC*	acc aga gac att gcc ttc c	467	94˚C 5 min	94˚C 30 s	53˚C 40 s	72˚C 45 s	72˚C 10 min	[38]
	ttc tgc tcg ccg cta ttc g							
*Salmonella sopB*	tca gaa gRc gtc taa cca ctc	517	94˚C 5 min	94˚C 30 s	58˚C 40 s	72˚C 45 s	72˚C 10 min	
	tac cgt cct cat gca cac tc							
*Salmonella avrA*	CCT GTA TTG TTG AGC GTC TGG	422 bp	94˚C 5 min	94˚C 30 s	58˚C 40 s	72˚C 45 s	72˚C 10 min	[38]
	AGA AGA GCT TCG TTG AAT GTC C							

A 25 µl reaction comprising 12.5 µl of EmeraldAmp Max PCR Master Mix (Takara, Japan), 1 µl of each primer at a concentration of 20 pmol, 4.5 µl of water, and 6 µl of DNA template was used to use the primers. The applied biosystem 2720 heat cycler was used to carry out the process.

The PCR products were separated by electrophoresis employing gradients of 5V/cm on a 1.5% agarose gel (Applichem, Germany, GmbH) in 1 × TBE buffer at room temperature. 15 µl of each product was put into a gel slot for gel analysis. The fragment sizes were measured using a gelpilot 100 bp ladder (Qiagen, gmbh, Germany) and a gene ruler 100 bp ladder (Fermentas, Germany). A gel documentation system (Alpha Innotech, Biometra) took pictures of the gel, and computer software was used to analyze the data.

#### Detection of the *Escherichia* coli genes

All *E. coli* isolates were subjected to PCR assay to detect the genes chuA, yjaA and tspE4C2 [31], luxS [32], Vt2e [33], astA [34], CFA/I [35] and groES [36] was detected using specific primers. Positive control DNA samples were incorporated into each reaction.

#### Detection of the *salmonella*’s genes

All *salmonellae* isolates were subjected to PCR assay to detect the invA [37], sopB, avrA and bcfC [38].

### PCR-DNA sequencing of immune, antioxidant and gastrointestinal (GIT) health genes

Using the Gene JET whole blood genomic DNA extraction kit and following the manufacturer's instructions, the genomic DNA was extracted from whole blood (Thermo scientific, Lithuania). The basis of this reaction is the use of Proteinase K to digest the blood samples in the provided Lysis Solution or Digestion Solution. After that, the lysate is combined with ethanol and put onto the purification column, which is where the DNA attaches itself to the silica membrane. Washing the column with the prepared wash buffers successfully removes impurities. Next, using the Elution Buffer, genomic DNA is eluted in an environment with low ionic strength.

DNA that was high quality, pure, and concentrated was tested by Nanodrop. The absorbance of the sample was read at 260 and 280 nm wavelength. The OD260/OD280 ratio for pure DNA is ≥ 1.8. Agarose gel electrophoresis was also used to detect DNA fragments after extraction.

Due to the fact that, the most prevalent and important commercially causing agents of diarrhea are bacteria; immune (*SELL, JAK2, SLC11A1, IL10, FEZF1, NCF4, LITAF, SBD2, NFKB, TNF-α, IL1B, IL6, LGALS,* and *CATH1*), antioxidant (*SOD1, CAT, GPX1, GST, Nrf2, Keap1, HMOX1*, and *NQO1*), and GIT health (*CALB1, GT*, and *MUC2*) gene segments were amplified by PCR. The primer sequences were created in accordance with the *Ovis aries* sequence that was published in PubMed. Table 3 displays the primers utilized for the amplification.

The polymerase chain reaction mixture was prepared in a thermal cycler with a final volume of 100 μL. 5 μL DNA, 68 μL H2O (d.d water), 25 μL PCR master mix (Jena Bioscience, Germany), and 1 μL of each primer were included in each reaction volume. For eight minutes, the reaction mixture was exposed to an initial denaturation temperature of 94 °C. Thirty cycles of denaturation at 94 °C for one minute, annealing temperatures for 45 s (as indicated in Table 2), extension at 72 °C for 45 s, and a final extension at 72 °C for eight minutes were the cycling protocol. Agarose gel electrophoresis was used to find representative PCR analysis results, and samples were stored at 4 °C. After that, a gel documentation system was used to visualize the fragment patterns under UV light.

**Table 2:** Forward and reverse primer sequence, length of PCR product and annealing temperature of investigated genes used in PCR-DNA sequencing

Gene	Forward	Reverse	Annealing temperature (°C)	Length of PCR product (bp)
SELL	5′-GTGACTCAGTGTGTGCCTTTGGA -3′	5′-TCACTCTCTTCGTTTATTGAGA-3′	62	410
JAK2	5′-GGTTTCGGAAGCAGGCAAGGCA -3′	5′-TCTATATGGAAGACATGGTTG -3′	60	420
SLC11A1	5′-TGCCCGGCACGCCAGCCACTGA-3′	5′-CTTGACTAGGGAGGAATGCAGGT-3′	60	840
IL10	5′-AGAGCGTCCGCCATGCCCAGCAG-3′	5′-TCACAGAGAAGCTCAGTAAAT-3′	58	747
FEZF1	5′-AGGTCTGTGACAAGCAGGGAAC-3′	5′-CTGCAGCGGCAGCGCGTCGCG-3′	60	495
NCF4	5′-AGAGGCAGCTCCTGGGGACTC-3′	5′-CCTCATGAGCTCCAGCAAGTC-3′	60	951
LITAF	5′-AGTCCGCCCCCCAGCTCCATCTG-3′	5′-GGAGACACGAGACCACCAAGGTA-3′	64	660
SBD2	5′-TTTGAAGGGTGGTGGCTCTAGGT-3′	5′-TCAGCTTCCAGACAGACGCTGAC-3′	58	960
NFKB	5′-CTCTGAATCATACAATGTTTAGT-3′	5′-ACACATCCAGCTGTCCTGTCCA-3′	60	540
TNF-α	5′-CCACCTCAGTTACCTTATTAT-3′	5′- ATGTCAAGTTCTAGGTGAGATCT -3′	60	323
IL1B	5′-CTACAGGTCAGTGGGAAAATTGA-3′	5′- CCATCAACCTCAAATAACAGCT-3′	62	802
IL6	5′-AAAGATCGCAGGTCTAATAACC-3′	5′- AACAGCCTAAACATATAAATACAAT-3′	58	552
LGALS	5′-ATGGACTCCTTGCCGAACCCCTA-3′	5′-TTATAACGTATCCACTGAAGTCA-3′	60	414
SOD1	5′-CATCCGCTTCGAGGCAAAGGGA-3′	5′-GGCAATTCCAATTACACCACA-3′	64	400
CAT	5′-ATGGCTTTTAATCCTACTTTCCT-3′	5′-ATTAATTCAAAGCAGGAATAAAC-3′	64	360
GPX1	5′-GTTCTTCAAGTCCCCAGCTCAC-3′	5′-TTCTACTCACTTCTGGCACTT-3′	60	865
GST	5′-GTGGGCAAGCCCAAGCTGCAC-3′	5′- GAGGCTTCCTCTGGCTGCCAG-3′	62	616
Nrf2	5′-CCGCTGCTCCTCTGCTCAAGA-3′	5′- GCTGCATGCAGTCATCGAAGT-3′	62	708
Keap1	5′-GGGCGTCCCGAGGCTAACCCC-3′	5′- ATGGACGCGGTGTAGGCGAACT-3′	58	757
HMOX1	5′-CCCCTCTACTTCCCAGAGGAG-3′	5′-ACAGCTGGATGTTGAGCAGGA-3′	60	415
NQO1	5′-AGAGTGGCACTCTGCACTTCTG-3′	5′-GAGCCTAATCAGATGTTGCCA-3′	64	525
CATH1	5′-ATGGAGACCCAGAGGGCCAGC-3′	5′- TCAGATCCAGTAGCTTGAGGC-3′	60	548
CALB1	5′-AGGACTCCCCGGGACGCCTGAGC-3′	5′-CTTGTATGTTGGAATATTATTA-3′	62	932
GT	5′-CAGCACCACAGCTCCTCCTTCT-3′	5′- TATGGCCCCTGCGGAGCCCTAC-3′	62	485
MUC2	5′-TGTGTGTGCTGGAGCATGCCGAG-3′	5′- GCTCTCTGCAGCAGGAGCACCT-3′	64	600

*-SELL* Selectin L, *JAK2* Janus kinase, *SLC11A1* Solute carrier family 11 A1, *IL10* Interleukin 10, *FEZF1* FEZ Family Zinc Finger 1, *NCF4* Neutrophil cytosolic factor 4, *LITAF* Lipopolysaccharide Induced TNF Factor, *SBD2* β-defensin, *NFKB* Nuclear Factor Kappa B, *TNF- α* Tumor necrosis factor- alpha, *IL1B* Interleukin 1 beta, *IL6* Interleukin 6, *LGALS* Galectin, *SOD1* Superoxide dismutase 1, *CAT* Catalase, *GPX1* Glutathione peroxidase 1, *GST* Glutathione S transferase, *Nrf2* Nuclear factor-erythroid factor 2-related factor, *KEAP1* Kelch-like ECH-associated protein 1, *HMOX1* Heme Oxygenase 1, *NQO1* NAD(P)H Quinone Dehydrogenase 1, *CATH1* Cathelicidin, *CALB1* Calbindin 1, *GT* Gastrotroponin and *MUC2* Mucin 2

Prior to DNA sequencing, nonspecific bands, primer dimmers, and other contaminants were eliminated. As described by [39, 40] PCR purification kit (Jena Bioscience #pp-201 × s/Germany) was used to purify PCR products with predicted size (target bands) in accordance with manufacturer instructions. In brief, the DNA fragment of interest was excised from an agarose gel, placed in a micro-centrifuge tube, solubilized in binding buffer and applied to the column. The chaotropic agent in the binding buffer dissolves agarose, denatures proteins and promotes DNA binding to the silica membrane in the column. Impurities were removed with a simple wash step. Purified DNA was then eluted from the column with the elution buffer. The Nanodrop (Uv–Vis spectrophotometer Q5000/USA) was used for PCR product quantification in order to maximise product yield and guarantee sufficient concentrations and purity of the PCR products. [41]. ABI 3730XL DNA sequencer (Applied Biosystem, USA) was used to sequence DNA in both forward and reverse orientations on PCR products containing the target band in order to identify SNPs in the genes examined in both healthy and diarrheal lambs. This was done in accordance with the enzymatic chain terminator technique created by [42]. The sequencing reaction was performed with four different fluorescent labels identifying the dideoxynucleotides (ddNTPs), instead of the radioactive labels. These fluorophores were excited with two argon lasers at 488 and 514 nm, respectively when the respective bands passed the lasers during the electrophoresis. The specific emissions were detected and the data were collected for analysis.

Chroma 1.45 and Blast 2.0 tools were used to analyze the DNA sequencing data [43]. The PCR results of the genes under study and the reference sequences found in GenBank were compared, and differences were categorized as single-nucleotide polymorphisms (SNPs). The MEGA6 software program was used to analyze the differences in the amino acid sequence of the genes under investigation between the enrolled subjects based on data alignment from DNA sequencing [44].

### Quantitative real time PCR for immune, antioxidant and gastrointestinal (GIT) health genes

Using Trizol reagent and the manufacturer's instructions (RNeasy Mini Ki, Catalogue no. 74104), total RNA was extracted from lamb blood. The NanoDrop® ND-1000 Spectrophotometer was used to quantify and qualify the amount of isolated RNA. Every sample's cDNA was created in accordance with the manufacturer's instructions (Thermo Fisher, Catalogue no. EP0441). Using SYBR Green PCR Master Mix (2 × SensiFastTM SYBR, Bioline, CAT No: Bio-98002) and quantitative RT-PCR, the gene expression patterns of the genes encoding immunity, antioxidants, and GIT health were evaluated. SYBR Green PCR Master Mix (Quantitect SYBR green PCR kit, Catalogue no. 204141) was used to perform real-time PCR for the relative measurement of the mRNA level. Table 4 displays the primer sequences created in accordance with the *Ovis aries* nucleotide sequence for all investigated genes that was published in PubMed (https://www.ncbi.nlm.nih.gov/nuccore/?term=Sheep). Due to involvement of healthy and diarrheic lambs as two categories in our investigation, the chance for obtaining conflict results were reduced as reported by previous studies [40]. Therefore; one housekeeping gene (*ß. actin*) was employed as a constitutive control for normalization.

In all, 3 µl of total RNA, 4 µl of 5 × Trans Amp buffer, 0.25 µl reverse transcriptase, 0.25 µl of each primer, 12.5 µl 2 × Quantitect SYBR green PCR master mix, and 4.75 µl RNase free water made up the 25 µl reaction mixture. Using a thermal cycler, the final reaction mixture was subjected to the following protocol: reverse transcription for 30 min at 50 °C, initial denaturation for 10 min at 94 °C, 40 cycles of 94 °C for 15 s, annealing temperatures as indicated in Table 3, and 72 °C for 30 s. A melting curve analysis was carried out at the conclusion of the amplification stage to verify the PCR product's specificity. Using the 2^−ΔΔCt^ technique, the relative expression of each gene in each sample was determined in relation to the *ß. actin* gene using the following equations [45];

**Table 3:** Oligonucleotide primers sequence, accession number, annealing temperature and PCR product size of investigated genes used in real time PCR

Gene	Primer	Product length (bp)	Annealing Temperature (°C)	Accession number
SELL	F5′- GACTTGGGTGGGAACCAACA -3 R5′- CACTGAGTCACTGTGTAGCAGA -3′	189	60	XM_012187246.4
JAK2	F5′- ATGTGAGTGAGAGCCGAACC-3′ R5′- GACTGCTCTTACCCGGTGC—3′	125	59	XM_042243421.1
SLC11A1	F5- CATGTCAGGTGACACGGGTA -3′ R5′- CCAGCCTGAAGATCCGACTC—3′	246	62	NM_001009345.1
IL10	F5′- CTGTGCCTCTCCCCTAGAGT -3′ R5′- GCAGCTAGCTCCACAAGGAA—3′	237	60	NM_001009327.1
FEZF1	F5′- TGAGATGCCTGCCGATTGTT -3′ R5′- GCTCATGAATGTGGCGTGTG—3′	127	60	XM_027969116.2
NCF4	F5′- ACTGGCTACGCTGCTACTAC-3′ R5′- TCAGGGTTCGGGAAAGGTCC—3′	146	58	XM_042247241.1
LITAF	F5′- GCTCCATCTGCTCAGACCTC-3′ R5′- GGCCACCGTCTCTTCATAGG—3′	136	59	XM_015104081.3
SBD2	F5′-TAGGAAGTCTACAAGCCCTTCT-3′ R5′- CAGGAAACTTATCAAAGTCACA—3′	81	62	MW201968.1
NFKB	F5′- TTCTTCGTGGTCCTGTCTGC-3′ R5′- CTCATGGTTCCAGGGCACTT—3′	113	59	NM_001198545.1
TNF-α	F5′-GCCTTTGGGGACTTCTCTCC-3′ R5′- GCAGGAACACGGTTACAGGA—3′	109	58	AF283892.1
IL1B	F5′-CTGCTGCACTTCGGGGTAA-3′ R5′-TGACGTCAGGGTCTTAACCA-3′	94	58	DQ153000.1
IL6	F5′- TGGGACGTTTTAGAGGTGGC-3 R5′- GTCCTCGGGGTTATTCAGCC -3′	108	60	NM_001009465.2
LGALS	F5- TGCAGTCCTCAAACGAGTGG-3′ R5′- CCGCAGCTACTTCATCCGAA—3′	110	58	NM_001009392.1
SOD1	F5- ACCTGCAGTCTGTTTCCCTG-3′ R5′- TGTTCATCACCACGATGCCA—3′	171	60	KM597535.1
CAT	F5-TGATCATGGGTTCCACGTCC-3 R5-CACATTGCCCAGGTCTCCAA-3	139	60	NM_001145185.2
GPX1	F5′-CAGTAGGAGACAAACTCAATG-3′ R5′- ACGACTCTCTCAGGAATTCTC—3′	121	62	GQ204786.1
GST	F5′-CGAGGAGATCCTGAATTGCCTGA-3′ R5′- ACCTCGCACTTTTCGAAGAGC—3′	95	60	JF728302.1
Nrf2	F5′-TGGCTGCAGCCGGAGTGGAGTT-3′ R5′-TGGCAACGTAGTTGAGAATGGC-3′	162	64	AJ238319.1
Keap1	F5′-TCACAAGGGAACAGCACTGCAG-3′ R5′-AGCCTCCAAGCGGCTTGAATG-3′	129	64	NM_001314327.1
HMOX1	F5′-TGAGAGTATCGGAGGCTACGCA-3′ R5′- CGCTAGGCCTGGGTTCCGGCT—3′	95	62	XM_027969637.2
NQO1	F5′- CAGGGACCAGACCTTCACAG-3′ R5′- GCATAAAGCCCCACAGCAAC—3′	167	60	XM_027967703.2
CATH1	F5′- GGCAGTGGCTCCATGTACTC-3′ F5′- TAGATGGCCACCTCACATGC-3′	116	60	XM_012190046.4
CALB1	F5′- CACGGTGAAAGAGACCGTGT-3′ F5′- CACACTGTTTCACCAGCCCA-3′	89	58	AB973433.1
GT	F5′- CCGCTCTACGATGGCAGAAT-3′ F5′- AGCGTCGAAATGAAGCCAGA-3′	85	62	XM_004011846.5
MUC2	F5′- GAGACCGAGATGGAGACCGT -3′ F5′- CTCTGCAGTGTGGTGGTAGTT -3′	105	62	XM_004009033.4
ß. actin	F5′- CGTGCTGCTGACGGAGGCCCC-3′ R5′- GCACAGCCTGGATGGCCACATAC -3′	113	60	AF481159.1

*-SELL* Selectin L, *JAK2* Janus kinase, *SLC11A1* Solute carrier family 11 A1, *IL10* Interleukin 10, *FEZF1* FEZ Family Zinc Finger 1, *NCF4* Neutrophil cytosolic factor 4, *LITAF* Lipopolysaccharide Induced TNF Factor, *SBD2* β-defensin, *NFKB* Nuclear Factor Kappa B, *TNF- α* Tumor necrosis factor- alpha, *IL1B* Interleukin 1 beta, *IL6* Interleukin 6, *LGALS* Galectin, *SOD1* Superoxide dismutase 1, *CAT* Catalase, *GPX1* Glutathione peroxidase 1, *GST* Glutathione S transferase, *Nrf2* Nuclear factor-erythroid factor 2-6666factor, *KEAP1* Kelch-like ECH-associated protein 1, *HMOX1* Heme Oxygenase 1, *NQO1* NAD(P)H Quinone Dehydrogenase 1, *CATH1* Cathelicidin, *CALB1* Calbindin 1, *GT* Gastrotroponin and *MUC2* Mucin

ΔCt for sample = Ct for sample-Ct for the reference (*β. actin*) gene; ΔΔCt = ΔCt for sample- ΔCt for control.

### Biochemical, immunological and antioxidants parameters

According to the author`s guidelines, the obtained plasma and serum were used in evaluation of the following parameters: ELISA kits of MyBiosecure Company® were used for evaluation of serum pro-inflammatory cytokines (IL-1α, IL-1β, IL-6, TNF-α) and anti-inflammatory cytokine (IL-10), ELISA kits of IBL International Crop (canda)® were used for determination of plasma fibrinogen (Fb), serum amyliod A (SAA) and serum haptoglobin (Hp), turbidimetric method were used for measuring of serum caeruloplasmin (Cp) and serum transferrin (Tf) using Elabscience USA® kits, CLIA method were used for determination of Serum ferritin by Abnova® (Taipei) kits, serum concentrations of free radicals (nitric oxide (NO), malondialdehyde (MDA)), antioxidants (catalase (CAT), glutathione peroxidase (GPx), glutathione reductase (GR)), serum iron (SI), total iron capacity (TIBC) (Spectrophotometrically using commercial kits of Biodiagnostic company®), Serum complement-3 (C3) and complement-4 (C4) (ELISA technique using commercial kits New Test Company®), Immunoglobulins (Igs) serum concentrations (IgG, IgM, IgA) (Turbidmetrically using kits supplied by Biorex diagnostics®, UK), Serum hormonal assays (cortisol, insulin, TSH, T3, T4 and growth hormone (GH) (Chemiluminescence immunoassay (CLIA) using kits supplied by Diasorin®, Italy).

Transferrin saturation percent (Tf sat. %) and unsaturated iron binding capacity (UIBC) was calculated according to the next formulae:

$$\begin{array}{c}\text{Tf sat}.\%=\frac{\text{SI}}{\textrm{TIBC}}*100\\ \text{UIBC}=\text{TIBC}-\text{SI}\end{array}$$

### Statistical analysis

- Independent-samples T test was used to compare the measured variables means of DG to CG using SPSS program version 23. A difference was considerable significant at *P* < 0.05.
- All variables of DG and CG were presented as mean ± SD.
- Pearson's simple correlation test was used for determination of correlations between the genetic and immunological, hormonal, iron profile parameters SPSS program version 23. A difference was considerable significant at *P* < 0.05.
- Cut off points, sensitivity, specificity and likelihood ratios (LR) for the measured cytokines and APPs were estimated **in** DG compared to CG using graphed prism version 8 program.
- The positive predictive value (PPV), negative predictive value (NPV), accuracy rate and percentages of increase for them were calculated by the following equations:
  $$\begin{array}{c}\text{PPV}=\text{True positive}\div \text{Total positive}*100.\\ \text{NPV}=\text{True negative }\div \text{Total negative}*100. \\ \text{Accuracy rate}=\left(\text{True positive}+\text{True negative }\right)\div \text{Total population}*100.\\ \text{Percentage of increaese}=\left(\text{The mean value of the marker concentration in DG }-\text{The mean value of its concentration in CG}\right)\div \text{The mean value of its concentration in CG }\times 100.\end{array}$$

## Results

### Clinical findings

Clinically, there was a significant (*P* < 0.05) increase of body temperature, pulse and respiratory rates (39.8 ± 0.3 °C, 97.2 ± 0.1 Beats/min and 46.5 ± 0.6 Breaths/min), respectively in DG in relation to CG (38.9 ± 0.04 °C, 87 ± 0.9 Beats/min and 38.2 ± 0.8 Breaths/min), respectively (Table 4).

**Table 4:** Changes in temperature, pulse and respiratory rates, in diarrheic Barki lambs compared to control group (mean ± SE)

Parameters	Control lambs	Diarrheic lambs	*p*- value
Temperature (°C)	38.9 ± 0.04	39.8 ± 0.3*	0.02
Pulse rate (Beats/min)	87 ± 0.9	97.2 ± 0.1*	0.001
Respiratory rate (Breaths/min)	38.2 ± 0.8	46.5 ± 0.6*	0.001

^*^Statistically significant when *P* < 0.05

### Isolation of *E. coli* virulence gene

**Fig. 1:**
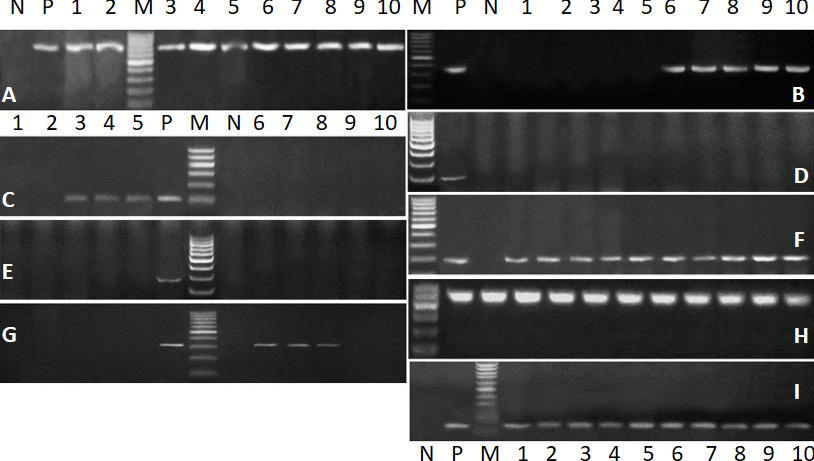
Representative gel electrophoresis of some virulence genes from *E. coli* strains isolated from sheep with diarrhea. **A**: *phoA* gene (genetic marker *of E. coli*), with expected band size 720 bp. **B**: *CFA/I* gene with band size of 364 bp. This gene was represented in 60% of isolated *E. coli* strains. **C**: *astA* gene with expected band size 110 bp. This gene was present in 50% of isolates. **D** & **E**: *yjaA* and *chuA* genes with expected band size 211 and 279 bp, respectively. These genes were not identified in any *E. coli* strain. **F**: *groES* gene was found in 100% of isolates with size 191 bp. **G**: *Vt2e* gene with size of 332bp. This gene was represented in 24% of isolated strains. (**H** & **i**: luxS and *tspE4C2 genes* of size 513 and 152 bp which were found in all strains

*E. coli* was identified genetically using *phoA* gene (genetic marker *in E. coli*), with primer as described in material and methods. *E. coli* which was isolated from all diarrheic lambs were 48/50 (96%), isolates carried gene *CFA/I* 30/50 (60%), *astA* 25\50 (50%), all samples did not carry genes *yjaA* and *chuA* (0%), groES gene (100%), *Vt2e ( Stx2e)* gene 12/50 (24%), *luxS and tspE4C2 genes* were found in all strains (Fig. 1 A—I).

### Isolation of *Salmonella* virulence gene

**Fig. 2:**
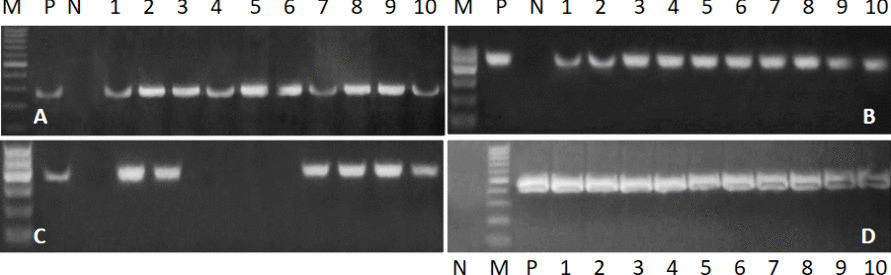
Representative agarose electrophoresis of some virulence genes from *Salmonella* spp. isolated from sheep with diarrhea. **A**: *invA* gene (genetic marker *of Salmonella*), with expected band size 284 bp. **B**: *sopB* gene with band size of 517 bp. This gene was represented in 92% of isolated *Salmonella* strains. **C**: *avrA* gene with expected band size 422 bp. Which was found in 78%? **D**: *bcfC gene with band size of 467 bp which present in 84% f isolated Salmenella strains*

All isolates were screened using PCR analysis for the presence or absence of 5 selected virulence genes, The invA was detected in 100% of the *Salmonella* strains; SopB 46/50 (92%), avrA 39/50 (78%) and bcfC 42/50 (84%) as shown in (Fig. 2 A -D).

### Genetic polymorphisms of immunity, antioxidant and GIT health genes

PCR-DNA sequencing of *SELL* (410-bp)*, JAK2* (420-bp)*, SLC11A1* (840-bp)*, IL10* (747-bp)*, LITAF* (660-bp)*, SBD2* (960-bp)*, TNF-α* (323-bp)*, IL1B* (802-bp)*, IL6* (552-bp)*, LGALS* (414-bp)*, CATH1* (548-bp), *SOD1* (400-bp), *GST* (616-bp), *Nrf2* (708-bp), *HMOX1* (415-bp), and *MUC2* (600-bp) genes found SNPs containing nucleotide sequence differences linked to either diarrhea resistance or susceptibility in Barki lambs. Meanwhile, *FEZF1* (580-bp), *NCF4* (951-bp), *NFKB* (540-bp), *CAT* (360-bp)**,***GPX1* (865-bp),* Keap1* (757-bp), *NQO1* (525-bp),* CALB1* (932-bp), *GT* (485-bp) genes caused a monomorphic configuration. All eleven SNPs were confirmed by nucleotide sequence variation of the genes under investigation between reference sequences in GenBank and those of the animals under study, resistant lambs, and afflicted lambs. Because of the exonic region alterations, the coding DNA sequences of the diarrheal lambs and healthy lambs were different (Table 5).

**Table 5:** Distribution of SNPs, type of mutation in immune and antioxidant genes in healthy and Diarrheic lambs

Gene	SNPs	Healthy	Diarrheic	Total	Type of mutation	Amino acid number and type
SELL SELL	T258C	29	-	29/100	Synonymous	86 F
JAK2	T350C	33	-	33/100	Non-synonymous	117 L to S
SLC11A1	A60T	23		23/100	Synonymous	20 S
	G231A	-	31	31/100	Synonymous	77 T
	T262C	-	26	26/100	Synonymous	88 L
	C381G	-	18	18/100	Synonymous	127 R
	C411A	26	-	26/100	Synonymous	137 G
	C449T	-	29	29/100	Non-synonymous	150 T to I
	A474G	-	34	34/100	Synonymous	158 L
	T792C	16	-	16/100	Synonymous	264 G
IL10	T399C	37	-	37/100	Synonymous	133 R
	G579C	17	-	17/100	Non-synonymous	193 R to S
LITAF	A285G	-	39	39/100	Synonymous	95 Q
SBD2	G430A	-	22	22/100	Non-synonymous	144 G to S
	G568A	31	-	31/100	Non-synonymous	190 A to T
	C593T	14	-	14/100	Non-synonymous	198 A to V
	C692T	41	-	41/100	Non-synonymous	231 S to F
	C874A	-	38	38/100	Non-synonymous	292 L to I
IL1B	A775C	-	13	13/100	Synonymous	259 R
TNF-α	C155T	-	42	42/100	Non-synonymous	52 T to M
IL6	C521T	18	-	18/100	Non-synonymous	174 T to I
LGALS	T26A	-	25	25/100	Non-synonymous	9 L to Q
	C136G	-	13	13/100	Non-synonymous	46 Q to E
	G139A	15	-	15/100	Non-synonymous	47 G to S
	C180G	36	-	36/100	Non-synonymous	60 I to M
	A193G	21	-	21/100	Non-synonymous	65 T to A
	C204T	30	-	30/100	Synonymous	68 D
	G215A	-	27	27/100	Non-synonymous	72 G to E
	C223G	16	-	16/100	Non-synonymous	75 Q to E
	C229G	43	-	43/100	Non-synonymous	77 L to V
	G231T	31	-	31/100		
	A233G	-	39	39/100	Non-synonymous	78 H to R
	G240C	-	23	23/100	Non-synonymous	80 E to D
	G247A	14	-	14/100	Non-synonymous	83 V to M
	C259T	-	25	25/100	Non-synonymous	87 P to S
	A272G	-	19	19/100	Non-synonymous	91 Q to R
	A314G	36	-	36/100	Non-synonymous	105 N to S
	A371T	-	25	25/100	Non-synonymous	124 D to V
SOD1	G76A	13	-	13/100	Non-synonymous	26 V to I
	G309A	31	-	31/100	Non-synonymous	103 K
	C370T	-	21	21/100	Non-synonymous	124 P to S
GST	C30T	27	-	27/100	Synonymous	10 N
	T56G	-	25	25/100	Non-synonymous	19 L to R
	T57G	-	33	33/100		
Nrf2	C190A	36	-	36/100	Synonymous	64 R
	C549G	21	-	21/100	Non-synonymous	183 H to Q
	G621C	14	-	14/100	Non-synonymous	207 Q to H
HMOX1	C112T	-	27	27/100	Non-synonymous	38 R to C
	T297C	-	36	36/100	Synonymous	99 S
CATH1	C264T	-	41	41/100	Synonymous	88 T
MUC2	T515C	25	-	27/100	Non-synonymous	172 M to T

*-SELL* Selectin L, *JAK2* Janus kinase, *SLC11A1* Solute carrier family 11 A1, *IL10* Interleukin 10, *FEZF1* FEZ Family Zinc Finger 1, *NCF4* Neutrophil cytosolic factor 4, *LITAF* Lipopolysaccharide Induced TNF Factor, *SBD2* β-defensin, *NFKB* Nuclear Factor Kappa B, *TNF- α* Tumor necrosis factor- alpha, *IL1B* Interleukin 1 beta, *IL6* Interleukin 6, *LGALS* Galectin, *SOD1* Superoxide dismutase 1, *CAT* Catalase, *GPX1* Glutathione peroxidase 1, *GST* Glutathione S transferase, *Nrf2* Nuclear factor-erythroid factor 2-related factor, *KEAP1* Kelch-like ECH-associated protein 1, *HMOX1* Heme Oxygenase 1, *NQO1* NAD(P)H Quinone Dehydrogenase 1, *CATH1* Cathelicidin. *CALB1* Calbindin 1, *GT* Gastrotroponin and *MUC2* Mucin 2

*-A* Alanine, *C* Cisteine, *D* Aspartic acid, *E* Glutamic acid, *F* Phenylalanine, *G* Glycine, *H* Histidine, *I* Isoleucine = K = Lysine, *L* Leucine, *M* Methionine, *N* Asparagine, *P* Proline, *Q* Glutamine *R* Argnine, *S* Serine, *T* Threonine; and *V* ValinE

### Gene expression pattern of immune, antioxidant and GIT markers

**Fig. 3:**
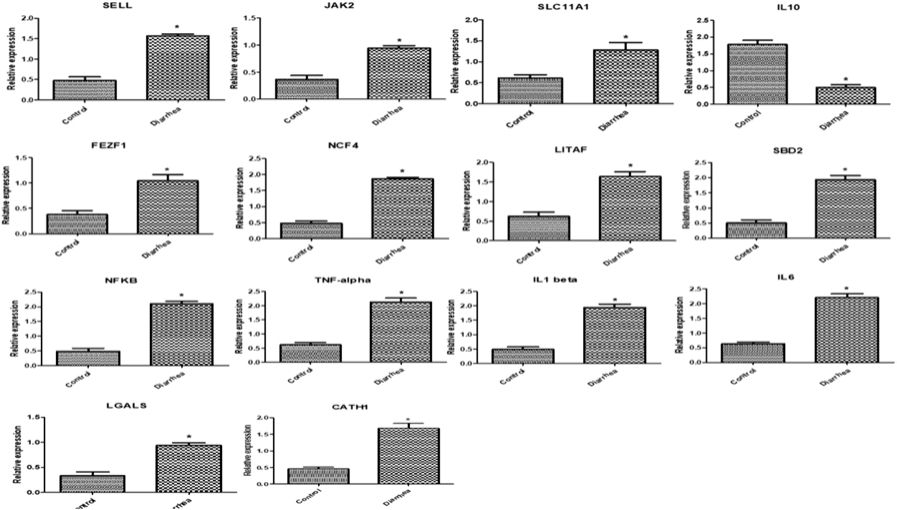
Differential transcript levels of immune genes between healthy and lambs with diarrhea. The symbol * denotes significance when *P* < 0.05

**Fig. 4:**
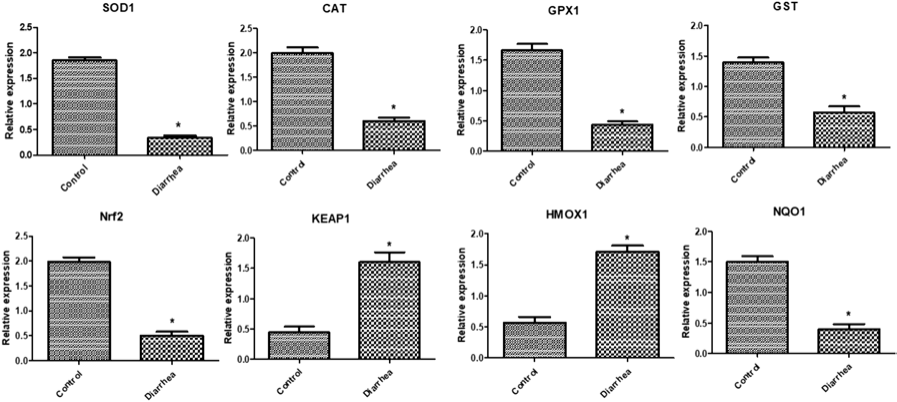
Differential transcript levels of antioxidant genes between healthy and lambs with diarrhea. The symbol * denotes significance when *P* < 0.05

**Fig. 5:**
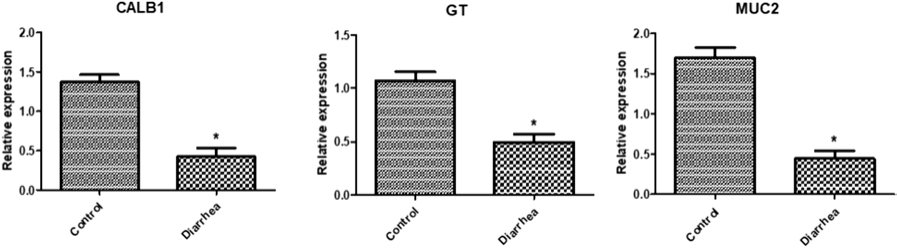
Differential transcript levels of intestinal health genes between healthy and lambs with diarrhea. The symbol * denotes significance when *P* < 0.05

Gene expression profile of immune, antioxidant and GIT markers was depicted in Figs. 3, 4 and 5. Levels of *SELL, JAK2, SLC11A1, FEZF1, NCF4, LITAF, SBD2, NFKB, TNF-α, IL1B, IL6, LGALS, CATH1*, *Keap1*, and *HMOX1* genes expression were considerably more abundant in diarrheal lambs than in resistant ones. *IL10, SOD1*, *CAT***,***GPX1*, *GST*, *Nrf2*, *Keap1*, *HMOX1*, *NQO1, CALB1*, *GT* and *MUC2* genes were significantly down-regulation in diarrheic lambs than resistant ones. *CAT* is the most up regulated gene (1.985 ± 0.11) while *galectins* is the most down- regulated gene (0.33 ± 0.07) in healthy Barki lambs. *IL6* is the most up-regulated gene (2.19 ± 0.0.12) while *SOD1* is the most down-regulated gene (0. 34 ± 0.03) in diarrheic Barki lambs.

### Hematological, biochemical and immunological profile

Complete blood count of DG demonstrated a significant (*P* < 0.05) erythrocytosis (marked by the significant (*P* < 0.05) raises in RBC, Hb, PCV and MCV) accompanied by significant neutrophilic leukocytosis (*P* < 0.05) and severe (*P* < 0.05) lymphopenia (Table 6).

**Table 6:** Hematological parameters of diarrheic lambs compared to control lambs

Parameters	Control lambs	Diarrheic lambs	*P* value
WBC(× 109/L)	10.10 ± 0.80	14.30 ± 0.70*	0.001
RBC (× 1012/L)	9.90 ± 0.08	11.20 ± 0.20*	0.001
Hb (g/dl)	12.20 ± 0.10	14.80 ± 0.70*	0.001
PCV%	44.30 ± 0.30	51.00 ± 0.50*	0.001
MCV (fL)	42.20 ± 0.4	46.50 ± 0.70*	0.001
MCH (pg)	13.10 ± 0.4	12.10 ± 0.10	0.064
MCHC (g/dl)	29.00 ± 1.20	27.50 ± 1.20	0.540
Lymphocytes (× 109/L)	4.10 ± 0.10	3.6 ± 0.06*	0. 001
Neutrophils (× 109/L)	6.00 ± 0.40	9.2 ± 0.30*	0. 001

^*^Statistically significant when *P* < 0.05

Table (7) showed that diarrhea in lambs was associated with a strong innate immune response, expressed as a significant (*P* < 0.05) increase in the pro-inflammatory cytokines (IL-1α, IL-1β, IL-6, TNF-α), acute phase proteins (Fb, SAA, Hp, Cp) and free radicals (NO, MDA) concentrations in DG when compared to CG. In contrast, the anti-inflammatory (IL-10), antioxidants (CAT, GPx, GR), C3 and C4 levels significantly (*P* < 0.05) decreased in DG in relation to CG. While, the significant (*P* < 0.05) hyperimmunoglobulinemia in DG (in relation to CG) referred to a strong humeral immune response. In parallel, the hormonal assays showed a significant (*P* < 0.05) elevation in cortisol, growth hormone concentrations in DG when compared to CG. Meanwhile, TSH concentrations non-significantly (*P* ≥ 0.05) changed and the insulin, T3 and T4 assays significantly (*P* < 0.05) depleted in DG in relation to CG. Conversely, the iron profile clarified a significant (*P* < 0.05) decline in SI, transferrin, Tf sat. % and a significant (*P* < 0.05) increase in TIBC, UIBC and ferritin between DG and CG.

**Table 7:** Immunological parameters, APPs, hormonal assays and iron profile of diarrheic lambs compared to control lambs (means ± SD)

Parameters	Control lambs	Diarrheic lambs	*P* value
IL-1α (Pg/ml)	24.05 ± 3.34	90.50 ± 1.78^*^	0.001
IL-1β (Pg/ml)	25.99 ± 2.96	101.80 ± 1.78^*^	0.001
IL-6 (Pg/ml)	24.63 ± 2.92	72.33 ± 6.64^*^	0.001
TNF-α (Pg/ml)	24.91 ± 2.98	71.26 ± 4.89^*^	0.001
IL-10 (Pg/ml)	103.70 ± 3.31	81.71 ± 1.05^*^	0.001
Fb (mg/dl)	122.01 ± 8.49	230.70 ± 12.42^*^	0.001
Cp (mg/dl)	2.30 ± 1.15	6.55 ± 0.07^*^	0.001
Hp (g/dl)	0.15 ± 0.02	2.54 ± 0.09^*^	0.001
SAA (mg/L)	2.32 ± 0.15	7.42 ± 0.41^*^	0.001
MDA (nmol/ml)	12.95 ± 1.14	26.89 ± 1.43^*^	0.001
NO (μmol/L)	26.80 ± 1.20	38.01 ± 2.90^*^	0.001
CAT (U/L)	412.25 ± 14.64	287.50 ± 5.74^*^	0.001
GPx (mU/L)	1015.45 ± 2.95	712.60 ± 3.56^*^	0.001
GR (ng/ml)	8.15 ± 0.62	6.10 ± 0.81^*^	0.001
C3 (mg/dl)	151.17 ± 5.29	126.09 ± 3.95^*^	0.001
C4 (mg/dl)	12.07 ± 0.60	9.60 ± 0.10^*^	0.001
IgG (mg/dl)	229.02 ± 19.74	452.22 ± 25.27^*^	0.001
IgM (mg/dl)	13.79 ± 1.43	54.35 ± 2.99^*^	0.001
IgA (mg/dl)	4.49 ± 0.83	10.59 ± 0.25^*^	0.001
Cortisol (μg/dl)	1.79 ± 0.16	6.91 ± 0.18^*^	0.001
Insulin (μIU/ml)	8.41 ± 0.15	7.41 ± 0.15^*^	0.001
T3(ng/ml)	1.74 ± 0.15	1.07 ± 0.02^*^	0.001
T4 (µg/ml)	0.85 ± 0.08	0.66 ± 0.03^*^	0.001
TSH (µIU/ml)	0.010 ± 0.002	0.010 ± .002	0.001
GH (ng/dl)	12.39 ± 1.47	16.51 ± 0.12*	0.001
SI (μg/dl)	106.89 ± 2.46	82.67 ± 2.02^*^	0.001
TIBC (μg/dl)	327.39 ± 2.16	354.31 ± 3.96^*^	0.001
UIBC (μg/dl)	220.50 ± 2.24	271.64 ± 4.32^*^	0.001
Transferrin(mg/dl)	124.65 ± 2.74	86.01 ± 2.90^*^	0.001
Tf sat. %	32.65 ± 0.66	23.33 ± 0.61^*^	0.001
Ferritin (ng/ml)	13.60 ± 1.05	20.01 ± 2.90^*^	0.001
^*^Statistically significant when *P* < 0.05			

Table (8) elucidated that both of estimated pro-inflammatory cytokines and APPs yielded sensitivity and NPV as 100% in DG. They also recorded high values of specificity, LR, PPV, accuracy rate but the highest specificity, LR, PPV, accuracy rate values were for IL-1α, IL-6, TNF-α, and Hp. Additionally, the percentages of increase for all of them were good but Hp percentage of increase was the best.

**Table 8:** Cut off points, sensitivity, specificity, LR, PPV, NPV, accuracy rate, and percentages of increase or decrease of interleukins and APPs in diarrheic lambs compared to control lambs

	IL-1α (Pg/ml)	IL-1β (Pg/ml)	IL-6 (Pg/ml)	TNF-α (Pg/ml)	IL-10 (Pg/ml)	Fb (mg/dl)	SAA (mg/L)	Hp (g/dl)	Cp (mg/dl)
Cut off	29.50	28.5	28.5	28.5	100.80	132.5	2.45	0.185	3.60
Sensitivity	100%	100%	100%	100%	100%	100%	100%	100%	100%
Specificity	90%	85%	90%	90%	80%	85%	80%	90%	80%
LR	10	6.67	10	10	5	6.67	5	10	5
PPV	90.9%	86.9%	90.9%	90.9%	83.3%	86.9%	83.3%	90.91%	83.3%
NPV	100%	100%	100%	100%	100%	100%	100%	100%	100%
Accuracy rate	95%	92.5%	95%	95%	90%	92.5%	90%	95%	90%
% increase or decrease	276.3%	291.6%	193.6%	186%	-26.9%	89%	219.8%	1593.3%	184.7%

### Correlation between gene expression pattern and serum profile of immune and antioxidant markers

The serum levels of IL1α, IL1β, IL6, FB, SAA, Hp, CAT, NO, GPx, IgG, insulin, T4, TSH, UIBC, ferritin, and TF were positively correlated with mRNA levels of *LITAF* (*r* = 0.999 and *p* = 0.04) and negatively correlated with mRNA levels of *IL6* (*r* = -0.998 and *p* = 0.03), *Nrf2* (*r* = -0.999 and *p* = 0.02) and *NQO1* (*r* = -1 and *p* = 0.009). Serum levels of IgM, and IgA were positively correlated with mRNA levels of *LITAF* (*r* = 1 and *p* = 0.003) and negatively correlated with mRNA levels of *NQO1* (*r* = -0.997 and *p* = 0.04). Serum levels of TNFα were negatively correlated with mRNA levels of *IL6* (*r* = -0.998 and *p* = 0.03) and *Nrf2* (*r* = -0.999 and *p* = 0.02). Serum levels of IL10 were negatively correlated with mRNA levels of *SBD2* (*r* = -0.997 and *p* = 0.04). Serum levels of GR were negatively correlated with mRNA levels of *FEZF1* (*r* = -1 and *p* = 0.002). Serum levels of C3 and cortisol were negatively correlated with mRNA levels of *IL6* (*r* = -0.999 and *p* = 0.02, *r* = -1 and *p* = 0.009), *Nrf2* (*r* = -1 and *p* = 0.009, *r* = -1 and *p* = 0.004) and *NQO1* (*r* = -1 and *p* = 0.007. *r* = -1 and *p* = 0.02). Serum levels of C4 were positively correlated with mRNA levels of *LITAF* (*r* = 0.999 and *p* = 0.03). Serum levels of T3 were positively correlated with mRNA levels of *LITAF* (*r* = 0.999 and *p* = 0.03) and MUC2 (*r* = 0.998 and *p* = 0.03).

## Discussion

It is thought that diarrhea is a significant source of economic loss due to its regular occurrence, especially in lambs, high rate of morbidity and mortality, slow growth, and veterinary expenses. This study's goal was to track changes in nucleotide sequences, gene expression, and indicators for oxidative stress and inflammation in the serum in lambs that had diarrhea. Another goal was finding the many *Salmonella* and *Escherichia coli* pathotypes and virulence genes that cause diarrhea.

In the current study, the diarrheic lambs suffered from off food, weakness, emaciation, dullness, depression, presence of soft feces, dehydration and pale mucous membranes with significant increase of body temperature, pulse rate and respiratory rate as compared with healthy ones. Our clinical findings were similar to that observed by [46–48] in lambs, [49] in buffalo calves and [50] in calves. The recorded anorexia, depression and dullness may be attributed to muscular weakness due to escape of intracellular potassium, hyperkalemia and hypoglycemia [51]**.** On the other hand, hyperthermia and pain signals could be attributed to infection and inflammation, hypothalamic thermoregulatory center motivation and the release pyrogenic substances such as prostaglandins [52].

In the present study, *E. coli* was isolated from 96% (48/50) of diarrheic lambs. This is in agreement with [53] who isolated *E. coli* from diarrheic goats, but higher than the percentage of *E. coli* reported by [54] who reported that the isolated of *E. coli* in chicken carcasses from Egypt was 92%. [55] isolation rate of *E. coli* from rabbits in Egypt was 86.7%, whereas [56] isolated *E. coli* in chickens from Iran by 76.3%, but unlike those reported by [57]**,** who observed *E. coli* in 70% of the diarrheic calves in Hawassa town, which could be attributed to small ruminants being more susceptible to diarrhea than calves, [58] isolated *E. coli* from diarrheic goats by 57.8%. Every country has a different method for isolating *E. coli* depending on the time of year, epidemiological factors, and animal flock management. The bacterial strain's virulence factors can enable colonization, growth, and survival in the face of the host's defensive mechanisms. [59].

From the lamb with diarrhea, we isolated the virulence gene arginine succinyl transferase (astA) from 25 out of 50 samples (50%) and the shiga toxin genes (Vt2e or Stx2e) from 12 out of 50 samples (24%). These results in diarrheal Slovak piglets between two and four weeks of age were in line with those of [60] in diarrheic Slovak piglets between 2 and 4 weeks old. The presence of gene encoding groES and luxS in all diarrheic samples (100%) was in agreement with other reports [61]. The gene CFA/I in (60%) of diarrheic lambs mediates bacterial adhesion to the intestinal cells and binds to the intestinal cell lining. This finding was consistent with that observed by [62].

The results of this investigation demonstrated the widespread distribution of virulence genes in *Salmonella.* Variations in *Salmonella* serovars' pathogenicity have been attributed to differences in their virulence factors. The detection rate of the invA gene in all studied isolates was consistent with recent reports in Egypt [63, 64] and throughout the world [65]. In contrast to our findings, [66] *Salmonella*-induced diarrhea in calves is extremely unusual, hence the inability to isolate *Salmonellae* from diarrheic calves may have been caused by this. However, *Salmonella* from diarrheic lambs was successfully isolated, and this was reportedly the first instance of diarrheic lambs in India. There could be a number of reasons for the discrepancies between the current study and the other earlier research, including variations in the study's duration spent in different locations, managerial variables, and cleanliness standards. An essential protein for epithelial cell invasion is encoded by the invA gene and is found in both the inner and outer membranes. These studies revealed this gene as a genetic marker for *Salmonella* serotype detection using PCR [67].

Based on the virulotyping results for tested *Salmonella*, PCR was performed using the five most important virulence genes..The invA was detected in 100% of the *Salmonella* strains; SopB 46/50 (92%), bcfC 42/50 (84%) and avrA 39/50 (78%). The avrA gene is only found in high numbers in serovars that could cause severe salmonellosis [68].

The gene avrA, which is linked to virulence, is found in SPI-1 and is present in most *Salmonella* strains [69]. AvrA is a multifunctional enzyme that stops the activation of the key proinflammatory (Nuclear Factor B) NF-B transcription factor and apoptosis through the c-Jun N-terminal Kinase (JNK) pathway [70]. It is phosphorylated in mammalian cells, and this phosphorylation needs the extracellular-regulated kinase (ERK) signalling pathway [71]. By stopping the breakdown of IB and -catenin, AvrA makes intestinal epithelial cells multiply [72] and tumors grow [73]. It speeds up the development of colon cancer caused by an infection by turning on the STAT3 signaling pathway [74]. Through the JNK pathway, *Salmonella'*s AvrA expression stabilizes the structure of tight junctions between intestinal epithelial cells and changes their work. *Salmonella*'s AvrA expression also makes it easier for bacteria to move and invade [75].

In our work gene sopB and bcfC have the highest recorded percent of tested virulence genes that's nearly similar to [65, 76]. The incorporation and assortment of such prophage-associated virulence genes may allow *Salmonella* to alter its behaviour and gain new characteristics. Fimbriae are also responsible for bacterial adherence to cells. They are a group of fimbrial determinants (including bcf) that are shared by *Salmonella* serovars and are responsible for host cell colonization [38]. The prophage-associated sopB gene was detected in 92% of the tested isolates. Additionally, numerous investigations have revealed the discovery of this gene in virtually all Salmonella strains isolated by [63, 64, 68] from food, humans and poultry, respectively.

In this context, PCR-DNA sequencing assessments for fragments of *SELL* (410-bp), *JAK2* (420-bp), *SLC11A1* (840-bp), *IL10* (747-bp), *LITAF* (660-bp), *SBD2* (960-bp), *TNF-α* (323-bp), *IL1B* (802-bp), *IL6* (552-bp), *LGALS* (414-bp), *CATH1* (548-bp), *SOD1* (400-bp), *GST* (616-bp), *Nrf2* (708-bp), *HMOX1* (415-bp), and *MUC2* (600-bp) genes showed differences in nucleotide sequences (SNPs) between diarrheal and resistant Barki lambs. Meanwhile, *FEZF1* (580-bp), *NCF4* (951-bp), *NFKB* (540-bp), *CAT* (360-bp), *GPX1* (865-bp), *Keap1* (757-bp), *NQO1* (525-bp), *CALB1* (932-bp), and *GT* (485-bp) genes elicited a monomorphic pattern. The monomorphic pattern between the enrolled animals may be attributable to PCR-DNA sequencing was analyzed on a conserved region, or exon, of the genes under investigation, which permits precise molecular characterization of genes and interprets physiological variations amongst livestock [77].

In our opinion, this is the first study to investigate SNPs in immunity (*SELL, JAK2, SLC11A1, IL10, FEZF1, NCF4, LITAF, SBD2, NFKB, TNF-α, IL1B, IL6, LGALS*, and *CATH1*), antioxidant (*SOD1, CAT, GPX1, GST, Nrf2, Keap1, HMOX1*, and *NQO1*) and GIT health (*CALB1, GT* and *MUC2*) genes as candidates for diarrhea resistance/susceptibility in Barki lambs. Comparing our results with a matched GenBank reference sequence revealed an interesting finding: the polymorphisms found in the genes under investigation are reported here for the first time. In Holstein dairy calves; PCR-DNA sequencing for the antioxidant (*Nrf2, Keap1*, and *HMOX1*) genes elicited nucleotide sequence variants between healthy and diarrheic individuals [78]. The candidate gene technique was employed to track the health of newborn animals experiencing diarrhea. For instance, [79] indicated no significant association of *CXCR1* SNPs with clinical intestinal diseases in dairy calves [80]. Also revealed a link between piglet diarrhea and a genetic variation in the swine leukocyte antigen-*DRA* gene. Moreover, [81] elicited Nramp1 gene polymorphism and its association with diarrhea in pigs. In goats, PCR-DNA sequencing was conducted for *TMED1, CALR, FBXW9, HS6ST3, SMURF1, KPNA7, FBXL2, PIN1, S1PR5, ICAM1, EDN1, MAPK11, CSF1R, LRRK1*, and *CFH* markers revealed nucleotide sequence variants between healthy and affected kids [40].

Real-time PCR was used in this investigation to measure the mRNA level of immunity (*SELL, JAK2, SLC11A1, IL10, FEZF1, NCF4, LITAF, SBD2, NFKB, TNF-α, IL1B, IL6, LGALS*, and *CATH1*), antioxidant (*SOD1, CAT, GPX1, GST, Nrf2, Keap1, HMOX1*, and *NQO1*) and GIT health (*CALB1, GT* and *MUC2*) genes in resistant and non-resistant Barki lambs to diarrhea. Our findings revealed that the expression pattern of *SELL, JAK2, SLC11A1, FEZF1, NCF4, LITAF, SBD2, NFKB, TNF-α, IL1B, IL6, LGALS, CATH1, Keap1*, and *HMOX1* genes expression was higher in diarrheic lambs than resistant ones. However, an opposite trend was noticed for *IL10, SOD1, CAT, GPX1, GST, Nrf2, Keap1, HMOX1, NQO1, CALB1, GT* and *MUC2* genes. Using a real-time PCR method, our work is the first to identify immunological, antioxidant, and GIT mRNA levels in lambs that are resistant to diarrhea and those who are not. Prior research investigated gene polymorphism in domestic animals using genetic markers such as RFLP and SNP [81]. Our study, however, was planned to address the shortcomings of earlier research by examining gene polymorphism using SNP genetic markers and gene expression. As a result, the methods by which the examined genes are regulated in both healthy and diarrheal lambs are well understood. To the best of our knowledge, there are very few published gene expression profiles of immunological, antioxidant, and GIT markers linked to a higher risk of diarrhea.

Regarding the ruminant gene expression profile, [82] showed that in goat peripheral blood mononuclear cells (PBMCs) infected with the bovine viral diarrhea virus-2, transcriptome analysis revealed differential expression of immune-related genes [81]. Further clarified that there were similarities between diarrheal and healthy newborn goats' TLR4 and downstream signaling pathways. It was also established that gene expression profile revealed that *TMED1, CALR, FBXW9, HS6ST3, SMURF1, KPNA7, FBXL2, PIN1, S1PR5, ICAM1, EDN1, MAPK11, CSF1R* and *LRRK1* were significantly up-regulated in diarrheic goats than resistant ones [40].

One of the most significant adhesion molecules found on polymorphnuclear cells is L-selectin, which is encoded by the *SELL* gene and is involved in neutrophil adherence to endothelium [83]. The primary mediators of intracellular signalling pathways are tyrosine kinases. The family of cytoplasmic tyrosine kinases known as JAK (Janus Kinase) is also a member of this group [84]. The primary role of JAK2 kinase is to participate in the signal transmission of hormones and cytokines, since it is predominantly involved in the signal pathways of STAT (Signal Transducer and Activator of Transcription) proteins [85]. Janus kinases catalyze the phosphorylation of proteins and indirectly start the transcription of target genes, which is why they are important for cell signalling at the cytokine level [86].

Trans-membrane protein Solute Carrier Family 11 Member 1 (SLC11A1) has been identified as one of the most well-known putative candidate genes that support innate immunity against several intracellular infections [87]. The zinc finger-like gene (*FEZL*) in the forebrain embryogenesis was found to be a QTL that affects mastitis resistance [88]. It also has an immunological function because it regulates neutrophil migration to the site of mammary gland infection, which is a crucial antibacterial activity [89]. One of the target genes of FEZL is semaphorine 5A (SEMA5A), which is a large family of secreted, membrane-associated proteins that are highly expressed [90]. Upon mastitis infection in cows, FEZL, a transcription factor, can stimulate SEMA5A and subsequently trigger the production of TNF-α and IL-8 [91]. In inflammatory circumstances, cytokines as NFKB, TNF-α, IFN-γ, IL1B, and IL-6 function as indirect indicators [92]. One of the primary pro-inflammatory cytokines in the immune response is TNF-α. TNF-α promotes the growth, development, and activity of numerous immune system cells in addition to other factors: Natural killer (NK) lymphocytes, T lymphocytes, B lymphocytes, and lymphokine-activated killer (LAK) cells [93]. Moreover, TNF-α induces the release of many other cytokines [94]. The *TNF-α* gene is found on chromosome BTA23q22 and consists of four exons and three introns [95]. While it is expressed in a variety of mammalian cell types, macrophages and monocytes have the strongest expression. Lipopolysaccharide (LPS) present in the bacterial cell wall stimulates TNF-α production in these phagocytic cells. When LPS stimulates macrophages, the *TNF-α* gene expression triples, mRNA levels rise by about 100 times, and protein secretion can increase by up to 10,000 times [96].

The new protein known as lipopolysaccharide-induced TNF-α factor (LITAF) binds to a crucial area of the *TNF-α* promoter and is implicated in inducing TNF-α production when LPS is present [97]. Vertebrates from many tissues and cell types, such as neutrophils and other leucocytes, epithelial cells, blood plasma, and urine, have been discovered to contain β-Defensins [98]. Defensins are secreted by neutrophils and monocytes (phagocytes) during inflammation as microbicidal agents [99].

According to reports, cytokine production and NFKB activation aid in the detection of microorganisms [100]. Interleukin-10, an anti-inflammatory cytokine, mainly functions as a feedback inhibitor of T cell responses and works in concert with other anti-inflammatory cytokines to regulate the host inflammatory response to microbial antigens [101]. The *NCF4* gene is a significant gene that codes for p40-phox, one of the NADPH oxidase subunits directly engaged in respiratory processes within cells [102]. This multi-subunit structure plays a substantial part in the defence mechanisms against microbial invasion. It has been demonstrated that this complex is translocated in the phagocytic cell membrane during the bacterial infection, where reactive oxygen species are created and the bacteria are managed [103].

Animal lectins belonging to the galectin family have a preference for β-galactosides [104]. Through lectin–carbohydrate interactions, they can interact with glycoproteins on the cell surface and in the extracellular matrix [104]. Galectins influence cell adhesion, stimulate migration, encourage cell development, and impact cell survival through this mechanism. They are present in a wide variety of cell and tissue types, and they have been linked to a number of different functions, including the encouragement of macrophage migration and involvement in both acute and chronic inflammation [105]. Cathelicidins, such as CATH1, are among the antibacterial weapons that leucocytes possess [106].

Antioxidants have three defensive mechanisms: they either detoxify or scavenge reactive oxygen species (ROS), prevent their synthesis, or sequester transition metals, which are the source of free radicals [107]. The body produces both enzymatic and non-enzymatic antioxidant defences, such as endogenous forms of glutathione peroxidase (GPx), glutathione S transferase (GST), catalase, and SOD [108]. As the main inducible defence against oxidative stress, the Keap1-Nrf2 stress response pathway controls the production of cytoprotective genes [109]. Under normal circumstances, cullin-based E3 ubiquitin ligase, which uses Keap1 as a substrate adaptor, ubiquitinates and proteasomally degrades Nrf2 to prevent its transcriptional activity [110]. This could explain the opposite pattern in gene expression that our investigation found for Keap1 and Nrf2.

In the heme catabolic pathway, heme oxygenase (HO) is the enzyme that limits the rate at which heme is broken down into equimolar amounts of biliverdin, free iron, and carbon monoxide (CO) [111]. Also referred to as a stress-responsive protein, it has a variety of protective roles against various stressors and is thought to possess anti-inflammatory, anti-apoptotic, anti-coagulant, anti-proliferative, and vasodilative qualities [112]. An antioxidant called NQO1 guards against the harmful oxidative and arylating effects of quines [113]. The process by which NQO1 detoxifies quinones involves reducing them directly to hydroquinones using two electrons, eliminating electrophilic quinones in the process, and avoiding the production of semiquinone radicals and reactive oxygen species through redox cycling processes [114].

Many tissues include the vitamin D-responsive gene calbindin, which helps transfer calcium across the cytosolic compartment of intestinal or renal cells in a way that prevents apoptotic cell death [115]. FABP6, sometimes referred to as ileal (gastrotropin) or fatty acid binding protein 6, is a protein that is encoded by the *FABP6* gene [116]. FABP6 functions are assumed to involve ileal fatty acid absorption, transport, and metabolism [117]. Entire mucosal surfaces are covered in mucins (MUC), which are highly O-glycosylated proteins [118]. They create a physical, chemical, and immunological barrier between the organism and its surroundings, which is why they are crucial to the GI tract defence mechanism [119].

Given that damaged tissues react more frequently to free radicals than healthy ones, it is possible that this accounts for the significant change in the expression pattern of immunological, antioxidant, and GIT markers in diarrheal lambs [120], and the intestine, which served as the primary location for a multitude of microbes, nutrients, and immune cell interactions, was very vulnerable to degradation [121]. The explanation could be that gastrointestinal pathogen invasions are strong oxidizing stimuli that trigger immune responses to counter pathogen invasions by triggering neutrophil and macrophage activity. This leads to an overabundance of ROS production and accumulation, which ultimately culminates in oxidative stress [122]. It is evident that TLR4 and the signaling pathways that it interacts with downstream play a critical role in triggering the release of inflammatory cytokines during bacterial infection. Diarrhea involves significant changes in the mechanism regulating the function of the gut barrier, potentially increasing the intestinal permeability to pathogenic bacteria [123]. Furthermore, ROS participates in intermicrobial competition [124]. As a result, we hypothesize that the diarrhea observed in these newborn lambs is primarily infectious in nature. Additionally, our Real-time PCR data provide credible proof that the diarrheal lambs were experiencing a significant inflammatory response.

Compared to healthy lambs, DG had significantly higher RBCs, Hb, PCV, MCV levels. These findings were similar to the findings of [125] in calves and [126] in lambs, but unlike to those reported by [49] who observed decreases in RBCs, MCH in diarrheic buffalo calves, [127] who showed non-significant changes in Hb values in diarrheic calves and [128] who revealed significant diminished PCV and MCH levels with non-statistical significance changes in Hb concentrations in diarrheic Arabian horse Foals when compared with healthy ones. This outcome could be explained by the oxidative damage to the intestines and the ensuing excessive fluid loss, hemoconcentration, and malabsorption of nutrients [129]. The DG observed neutrophilic leukocytosis may be related to the activation of pro-inflammatory cytokines, which cause the bone marrow to produce more neutrophils and release them into the host circulation to kill bacterial invaders during inflammation and infections [130]. On the other hand, lymphopenia in DG may result from the dwindling of lymphocytes and ileal follicles in Peyer's patches [131].

Concerning the immunological alterations, the DG showed a marked increase in the pro-inflammatory cytokines concentrations (IL-1α, IL-1β, IL-6, TNF-α) and a noticeable decline in the anti-inflammatory cytokine (IL-10) level. These outcomes were in line with what was observed in lambs by [132]. The immune response is usually described as a symphony, played with an amazingly organized orchestra. The maestro of this orchestra is the pro-inflammatory cytokines which are non-specifically stimulated by the external pathogens and destructed tissue to communicate and coordinate between the other symphony players (immune molecules) to initiate, propagate and maintain the inflammatory-immune response [133]. Anti-inflammatory cytokine (IL-10) works antagonistically against the pro-inflammatory cytokines in order to limit their action and its observed decreased levels in DG, led to the exacerbation of the pro-inflammatory cytokines` actions and subsequent other immunological (innate or humeral), hormonal and iron profile changes [132].

An additional element of the innate immune response is the acute phase proteins. These non-specific hepatic proteins are generated in response to pro-inflammatory cytokines [134]. In this study, the serum concentrations of APPs (Fb, SAA, HP, and Cp) were significantly elevated in lambs that had diarrhea. These results were comparable to those of adult buffalo and calves published by [135, 136]. They play a major role in maintaining bodily harmony and homeostasis as well as controlling microbial development until a particular immunity is formed. Besides, they have potent anti-inflammatory, anti-bacterial and anti-oxidant actions [133]. Furthermore, APPs could be useful as prognostic tools and facilitate treatment decisions [135].

In terms of the oxidative stress markers, the serum levels of free radicals (NO, MDA) significantly increased in the DG research, whereas the levels of antioxidants (CAT, GPx, GR) significantly reduced. This imbalance between the levels of free radicals and the activity of enzymatic antioxidants in DG led to the emergence of oxidative stress and the consequent oxidative intestinal damage in the disease animals [137]. Oxidative stress was widely reported before in different animals' diseases including enteritis [138]. Despite having originated as an immunological response, oxidative stress had a significant role in the etiology of the disease. In essence, many immune cells release free radicals in reaction to the pro-inflammatory cytokine triggers listed above. Essentially, they cause oxidation and damage to the pathogen's proteins, carbohydrates, and DNA through non-specific reactions. To shield the body from the damaging oxidative effects of these substances, the enzymatic antioxidants tightly neutralize and regulate their activity. According to the current research, oxidative stress and oxidative tissue damage were caused by free radicals that collected and outnumbered the ability of enzymatic antioxidants to neutralize them [138].

The complement system is an important player in the immune system orchestra. It binds the previous non-specific innate immunity with next specific humeral immunity [139]. Similar to the acute phase response and free radicals, it activated through the pro-inflammatory cytokines invigoration. It works in a cascade fashion to generate membrane attack complex, opsonins and chemoattractants in order to facilitate the invading organisms` elimination and destruction. The available data showed that DG's C3 and C4 levels had significantly decreased. This referred to the inflammatory process magnification in DG and the hyper-activation of the complement cascade and subsequent splitting and cleavage of C3 and C4 to eliminate the invading organism [140]. According to earlier research, proper complement system activation is required to resolve chronic intestinal inflammation; on the other hand, dysregulation or over-activation of the system may exacerbate intestinal inflammation [141]. However, in this investigation, immunoglobulin serum concentrations increased in DG. Prior research by [142] found hyperimmunoglobulinemia in diarrheatic lambs experimentally infected with Haemonchus contortus, but [143] reported hypoimmunoglobulinemia in diarrhea in nursing lambs. The distinct hyperimmunoglobulinemia in DG suggested a robust humeral immunity and was also linked to the pro-inflammatory cytokines that had previously been stimulated and enhanced the particular generation of B-lymphocytes to eliminate external pathogens [133].

When compared to healthy groups, the endocrine changes in DG in this study showed a considerable increase in serum levels of growth hormone and cortisol and a significant decrease in serum concentrations of insulin, T3, and T4. Similar hormonal changes have previously been linked to a variety of pediatric intestinal disorders [144]. These alterations may be related to hypoglycemia, which is typically associated with diarrhea, as a result of the anorexia and malabsorption associated with the pathophysiology of the disease [145]. In order to counteract this hypoglycemia and preserve the energy needed to sustain the immunological response, the endocrine system kicks in [146]. In order to prevent glucose from being absorbed by cells, the pancreatic islets produce less insulin and the pituitary gland secretes more growth hormone [130]. The adrenal gland stimulates the release of cortisol, which affects hepatic gluconeogenesis and glycogenolysis, promotes insulin resistance, and inhibits the production of catabolic hormones (T3 and T4) [146].

In this study, the iron profile of DG showed hypoferremia, hyperferritinemia, and hypotransferrinemia. Similar findings in lamb protozoal diarrhea have previously been noted [147, 148]. These alterations influenced the immune response in multiple ways. The earlier pro-inflammatory cytokines that have been activated aim to stop the growth of the invasive bacteria by lowering transferrin activity, increasing iron storage such as ferritin and hemosiderin, and releasing hepcidin to limit intestinal iron absorption [133]. Basically, this action is anti-microbial but in chronic cases it results in anemic changes in the diseased animals. The acute phase response is also involved in these alterations whereas ferritin is a positive acute phase reactant and transferrin is a negative acute phase reactant [133]. Additionally, the liberated free radicals attack and destroy the red blood cells leading to increase iron release and dependent ferritin formation [149]. Logically, the hypoferremia identified in DG was caused by the reduced intestinal absorptive surface brought on by diarrhea linked to the pathophysiology of the disease [138]. Following the hypoferremia previously noted, there is a noticeable increase in TIBC, UIBC, and a decrease in Tf sat %.

Table (8) highlighted the diagnostic importance of estimated pro-inflammatory cytokines and APPs, indicating their sensitivities as biomarkers for sheep enteritis, particularly emphasizing IL-1α, IL-6, TNF-α, and Hp due to their highest specificities, LRs, PPVs, and accuracy rates. Hp stood out as the top choice among these markers based on its percentage increase, surpassing other contenders' increase percentages [123, 140]. These findings are consistent with earlier research [135] that established the predictive and diagnostic use of APPs in a range of animal illnesses and their function in directing therapeutic choices. However, they differ in terms of the markers priority. This discrepancy may be due to differences in animal species, disease stage, severity, and etiology.

The current study examines a novel method for highlighting the importance of SNPs in immune, antioxidant, and intestinal health-related genes as genetic markers and risk factors for diarrhea resistance or susceptibility in Barki lambs. It also assesses the relationship between the gene expression pattern and the serum profile of immune and antioxidant markers in diarrhea lambs. According to these results, variations in these genes may serve as surrogate biomarkers for developing a successful treatment strategy for lambs with this kind of disease. Furthermore, the differing immunological, antioxidant, and GIT health-related gene expression patterns in diarrhea-resistant and non-resistant lambs may serve as a biomarker and reference tool for monitoring the health of the lambs. Furthermore, these findings present a viable way to reduce lamb diarrhea by selectively breeding animals according to genetic markers linked to innate immunity to infection. Thus, a wide range of factors must be considered in subsequent research to obtain a concrete conclusion.

## Conclusion

The results herein confirmed the presence of virulent genetic markers of pathogenic characteristics of *E. coli* (astA, Vt2e (Stx2e), CFA/I, *groES* and *luxS*) and *Salmonella* (*invA*, *SopB*, *bcfC* and *avrA*) in the diarrheic Barki lambs. In immunological, antioxidant, and intestinal health genes, differences in individual genomic regions and expression profiles were found between diarrhea healthy and resistant lambs. Lamb enteritis is characterized by a shift in the biochemical profile of inflammatory markers, which triggers both an innate and a humeral immune response. Changes in the iron profile markers and hormonal tests also occur concurrently with this immune response. These findings suggest that differences in these genes could act as stand-in biomarkers for creating an effective treatment plan for lambs suffering from this type of disease by predicting, preventing, and selecting resistant sheep. Future selection for diarrhea-resistant animals by the gene targets discovered here could also decrease economic losses afforded by animal breeders. As any trace changes could be detected by real time PCR approach, the variable immune, antioxidant, and gastrointestinal tract health-related gene expression patterns between diarrheic and resistant lambs may be used as a biomarker and reference tool to track the health of the lambs. Furthermore, by carefully breeding animals based on genetic markers associated with innate immunity to infection, our findings offer a practical means of reducing lamb diarrhea. A large number of investigated markers, a wide range of diverse sheep, and the study of etiological agents causing diarrhea should be acknowledged by further studies.

## Supplementary Information

Supplementary Material 1.

## Data Availability

The datasets for DNA sequence generated and/or analysed during the current study are available in the GenBank with accession numbers gb|OR900032|, gb|OR900033|, gb|OR900034|, gb|OR900035|, gb|OR900036|, gb|OR900037|, gb|OR900038|, gb|OR900039|, gb|OR900040|, gb|OR900041|, gb|OR900042|, gb|OR900043|, gb|OR900044|, gb|OR900045|, gb|OR900046|, gb|OR900047|, gb|OR900048|, gb|OR900049|, gb|OR900050|, gb|OR900051|, gb|OR900052|, gb|OR900053|, gb|OR900054|, gb|OR900055|, gb|OR900056|, gb|OR900057|, gb|OR900058|, gb|OR900059|, gb|OR900060|, gb|OR900061|, gb|OR900062|, gb|OR900063|.

## Abbreviations

C3: Complement-3
C4: Complement-4
CAT: Catalase
Cp: Caeruloplasminhaptoglobin
Fb: Fibrinogen
GPx: Glutathione peroxidase
GR: Glutathione reductase
Hb: Hemoglobin concentration
Igs: Immunoglobulins.
IL-10: Interleukin 10
IL-1α: Interleukin 1 alpha
IL-1β: Interleukin 1 beta
IL-6: Interleukin 6
MCH: Mean corpuscular hemoglobin
MCHC: Mean corpuscular hemoglobin concentration
MCV: Mean corpuscular volume
MDA: Malondialdehyde
NO: Nitric oxide
PCV: Packed cell volume
RBCs: Red blood cell count
SAA: Serum amyloid A
SI: Serum iron
Tf sat. %: Transferrin saturation percent
Tf: Transferrin
TIBC: Total iron capacity
TNF-α: Tumor necrosis factor alpha
UIBC: Unsaturated iron binding capacity
WBCs: Total leukocytic count
