# Supplementary material for: Nucleotide sequence variants, gene expression and serum profile of immune and antioxidant markers associated with bacterial diarrhea susceptibility in Barki lambs
Source: BMC Vet Res. 2024 Oct 11;20:462. doi: 10.1186/s12917-024-04288-1 (PMC11468138; doi:10.1186/s12917-024-04288-1)
Supplement: Supplementary file 1 — Supplementary Material 1. [file 12917_2024_4288_MOESM1_ESM.docx]

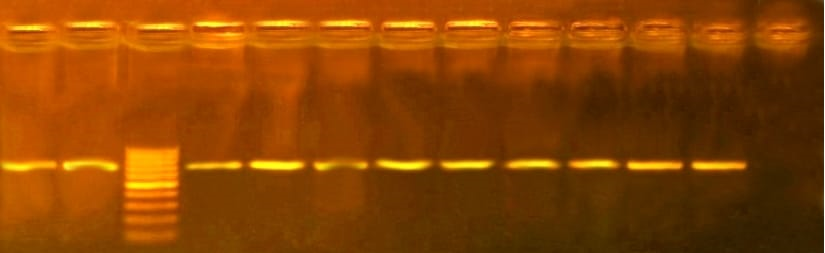


(A): *phoA* gene (genetic marker *of E. coli*), with expected band size 720 bp.


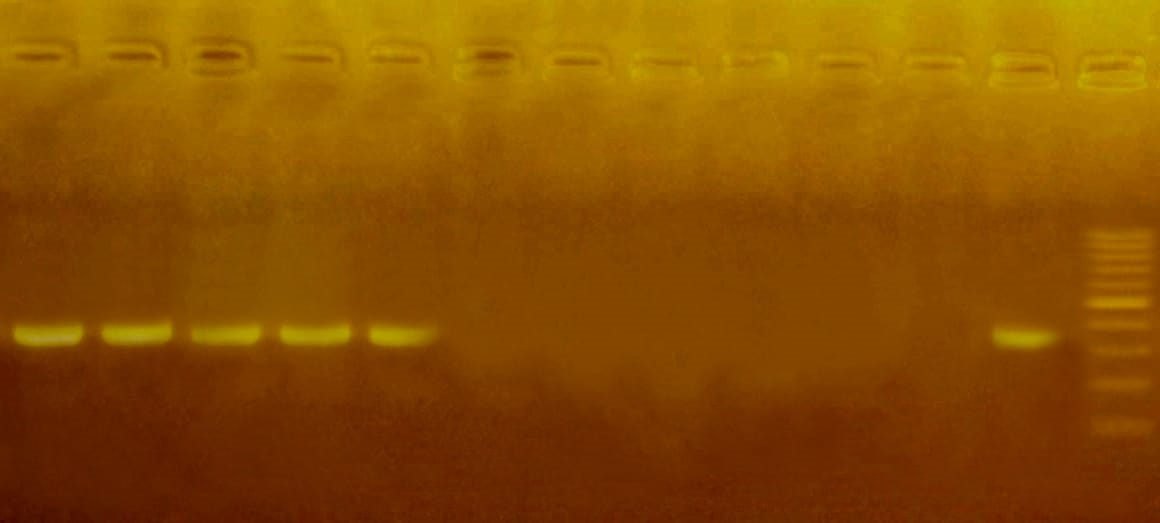


(B): *CFA/I* gene with band size of 364 bp.


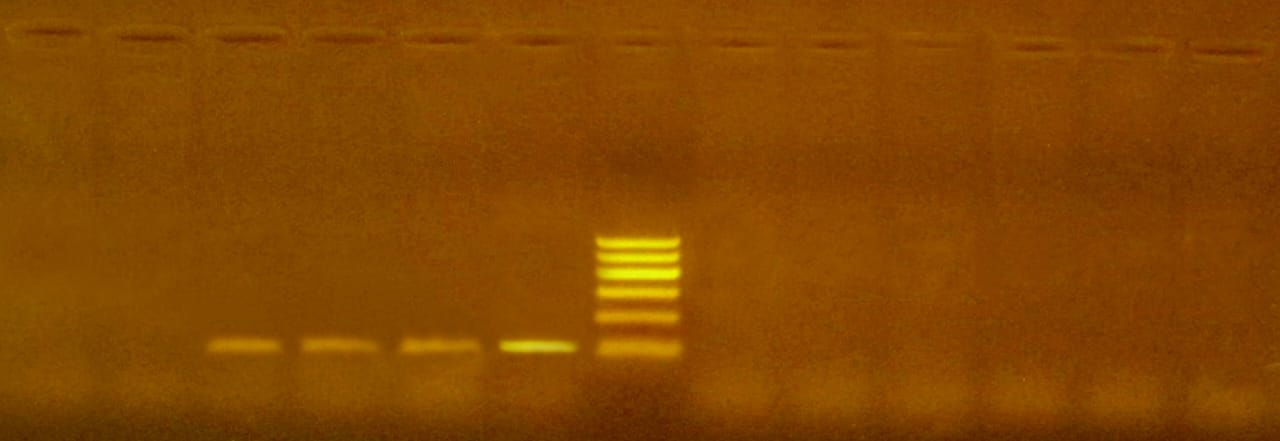


. (C): *astA* gene with expected band size 110 bp.


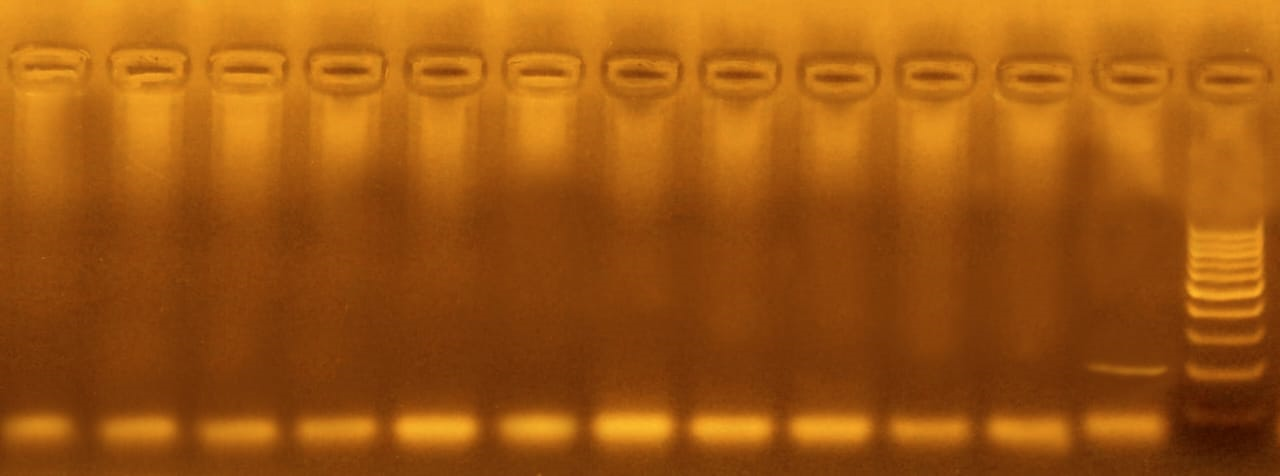


(D): *yjaA* gene with expected band size 211.


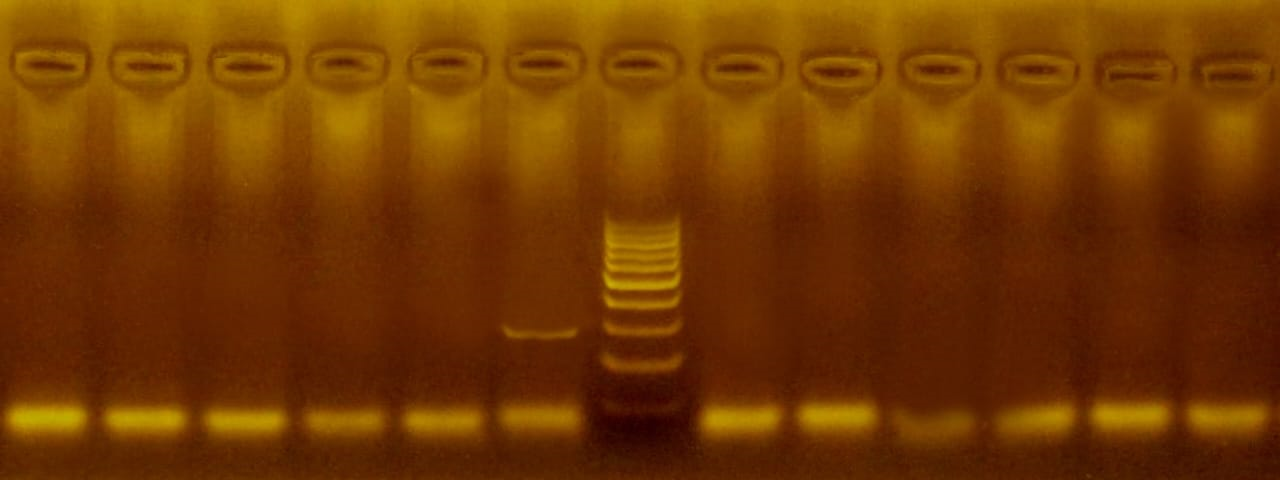


(E) *chuA* gene with expected band size 279 bp.


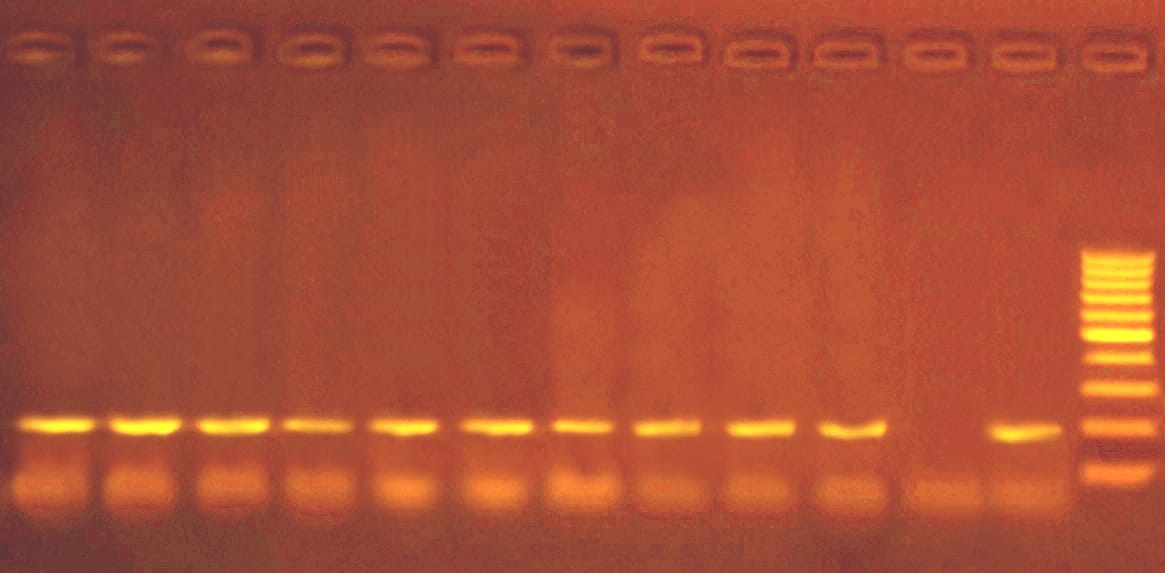


(F): *groES* gene was found in 100% of isolates with size 191 bp.


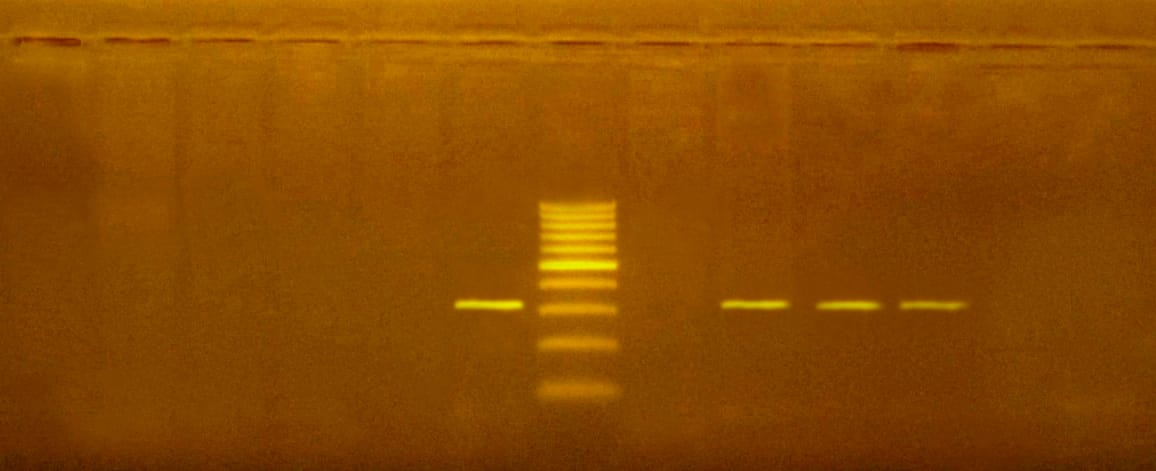


(G): *Vt2e* gene with size of 332bp.


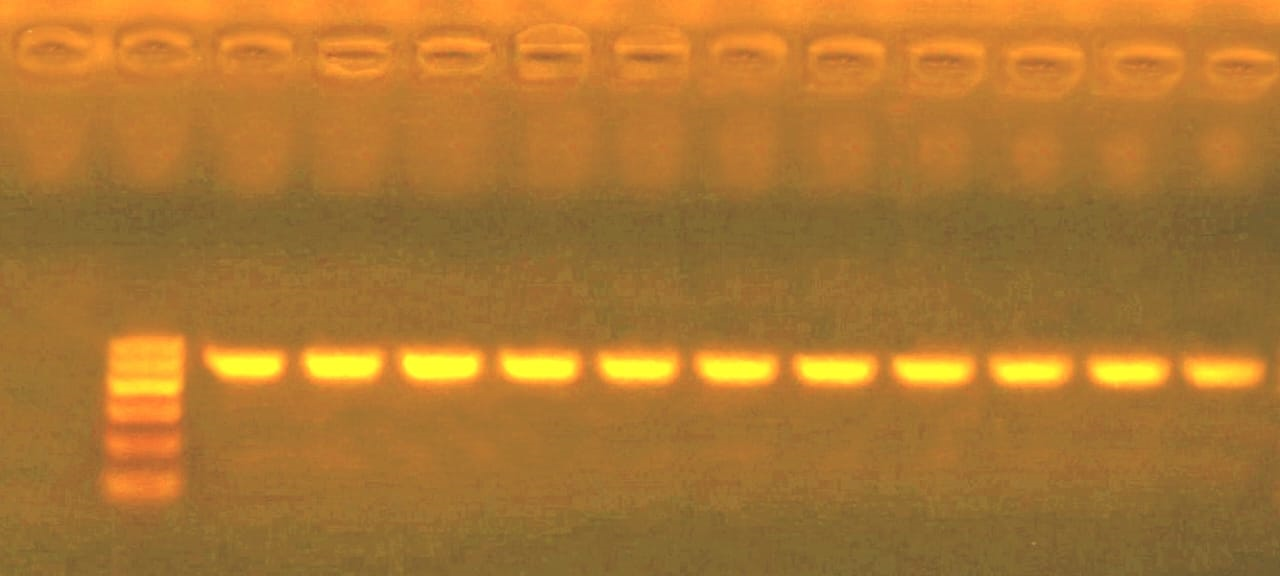


(H): luxS *gene* of size 513.


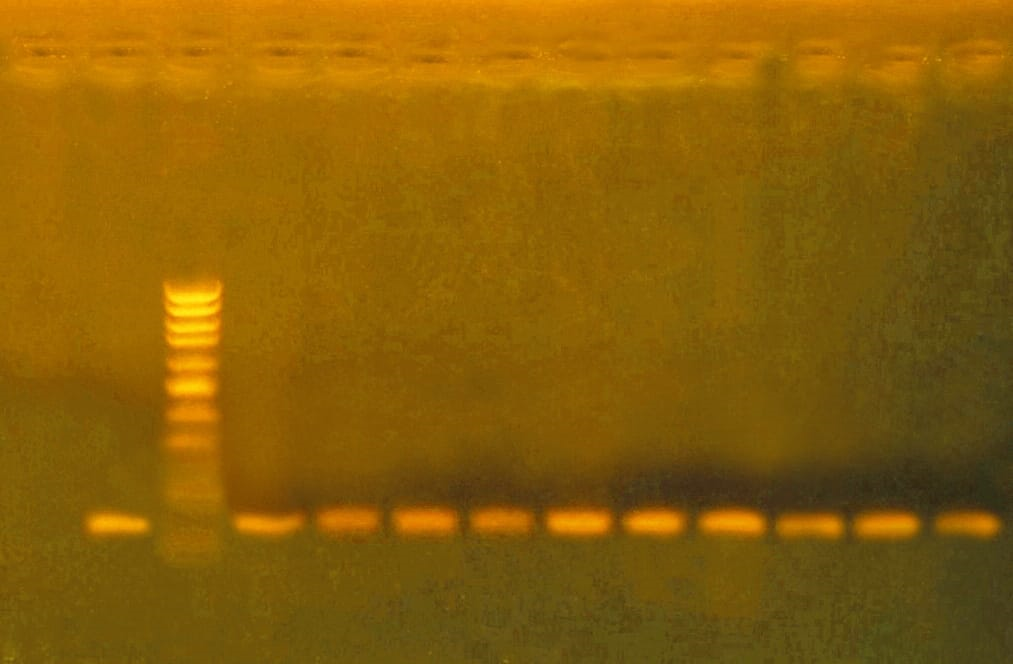


(i): *tspE4C2 gene* of size 152 bp.

**Figure 1.** Representative gel electrophoresis of some virulence genes from *E. coli* strains isolated from sheep with diarrhea. (A): *phoA* gene (genetic marker *of E. coli*), with expected band size 720 bp. (B): *CFA/I* gene with band size of 364 bp. This gene was represented in 60% of isolated *E. coli* strains. (C): *astA* gene with expected band size 110 bp. This gene was present in 50% of isolates. (D & E): *yjaA* and *chuA* genes with expected band size 211 and 279 bp, respectively. These genes were not identified in any *E. coli* strain. (F): *groES* gene was found in 100% of isolates with size 191 bp. (G): *Vt2e* gene with size of 332bp. This gene was represented in 24% of isolated strains. (H & i): luxS and *tspE4C2 genes* of size 513 and 152 bp which were found in all strains.


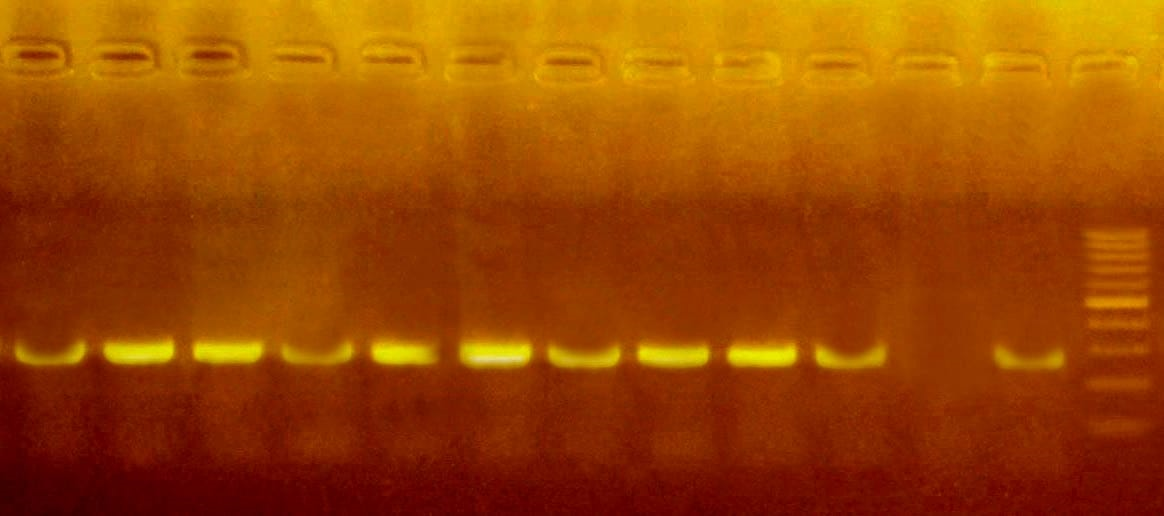


. (A): *invA* gene (genetic marker *of Salmonella*), with expected band size 284 bp


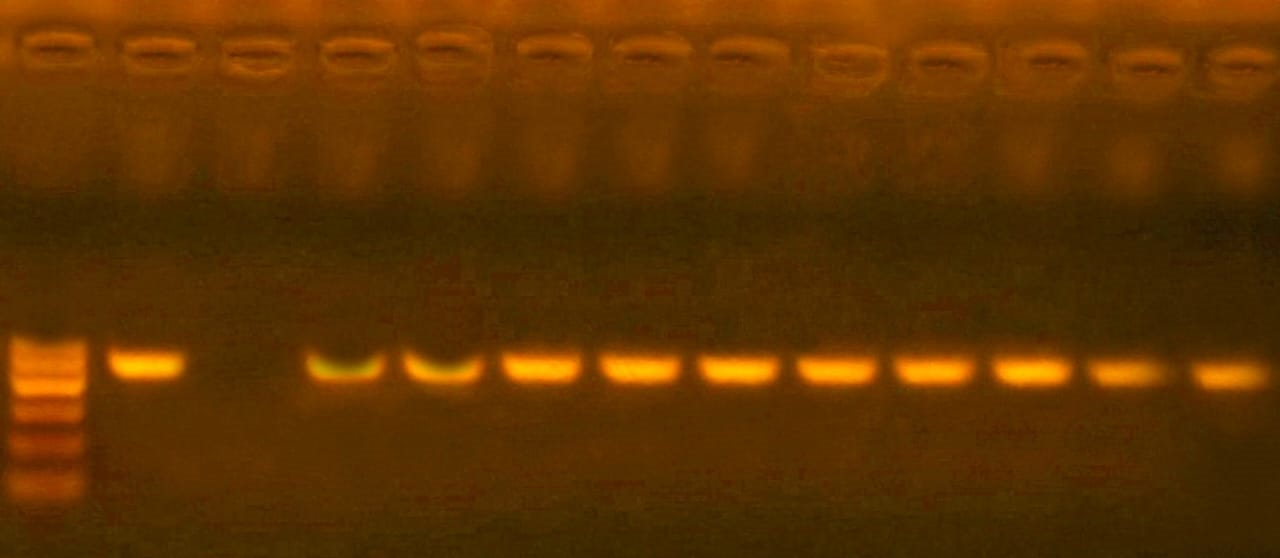


(B): *sopB* gene with band size of 517 bp.


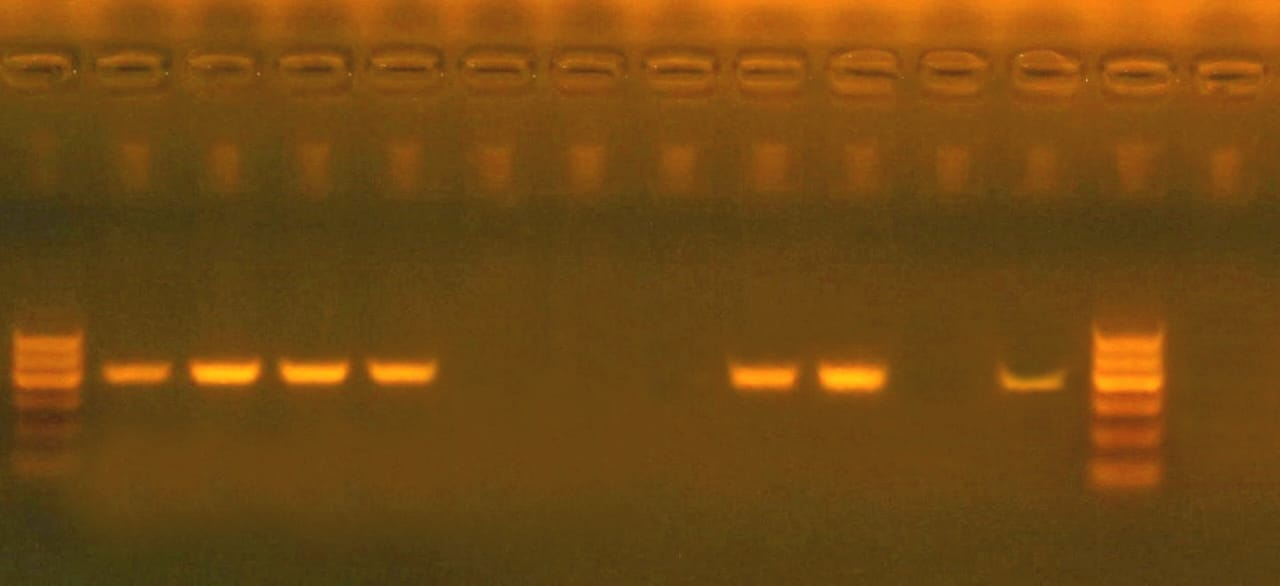


(C): *avrA* gene with expected band size 422 bp


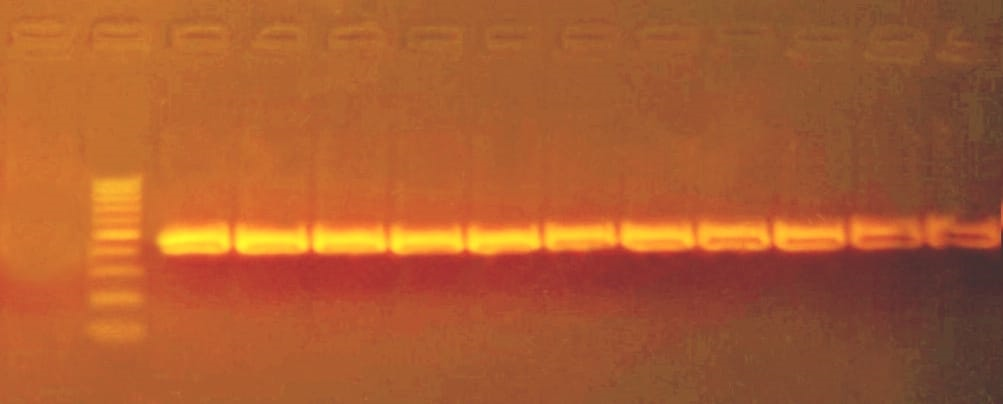


(D): *bcfC gene with band size of 467 bp*

**Figure 2.** Representative agarose electrophoresis of some virulence genes from *Salmonella* spp. isolated from sheep with diarrhea. (A): *invA* gene (genetic marker *of Salmonella*), with expected band size 284 bp. (B): *sopB* gene with band size of 517 bp. This gene was represented in 92% of isolated *Salmonella* strains. (C): *avrA* gene with expected band size 422 bp. Which was found in 78%? (D): *bcfC gene with band size of 467 bp which present in 84% f isolated Salmenella strains.*
